# Clinical practice guidelines and quality standards for early intervention in psychosis: an AGREE II appraisal and systematic review of service components

**DOI:** 10.3389/fpsyt.2026.1831668

**Published:** 2026-06-03

**Authors:** Pasquale Scognamiglio, Vassilis Martiadis, Giuseppe Diaspro, Luca Viacava, Anna Longobardi, Raffaele Rea, Valeria Iniziato, Pasquale Saviano, Silvestro La Pia

**Affiliations:** 1 Department of Mental Health, ASL Napoli 3 Sud, Torre del Greco, Italy; 2 Department of Mental Health, ASL Napoli 1 Centro, Napoli, Italy; 3 Department of Mental Health, ASL Salerno, Salerno, Italy; 4 Territorial Assistance Department, ASL Napoli 3 Sud, Torre del Greco, Italy

**Keywords:** AGREE II, clinical high risk for psychosis, clinical practice guidelines, early intervention in psychosis, first-episode psychosis, quality standards, systematic appraisal, youth mental health

## Abstract

**Background:**

Early intervention in psychosis (EIP) is a key component of youth mental health care, yet recommended models of care for individuals at clinical high risk for psychosis (CHR-P) and first-episode psychosis (FEP) remain heterogeneous across jurisdictions. No previous study has combined a formal AGREE II appraisal with a structured synthesis of core service components for EIP.

**Methods:**

We conducted a systematic review and AGREE II-based appraisal of international clinical practice guidelines (CPGs) and quality standards (QSs). MEDLINE (via PubMed), the Cochrane Library (CENTRAL), Web of Science, guideline repositories, and grey literature sources were searched to March 2025 in accordance with PRISMA 2020 guidance. Eligible documents were CPGs or QSs published in English or Italian from 2005 onward containing recommendations on the organization, assessment, and treatment of CHR-P and/or FEP. Methodological quality was appraised with AGREE II (inter-rater agreement assessed using ICCs); recommendations were extracted, and core components required endorsement by at least one third of relevant documents with at least one strong or mandatory recommendation. Twenty-six documents (24 CPGs and 2 QSs) were included. Methodological quality was moderate overall, with substantial variability across AGREE II domains: scores were generally strongest for scope and purpose, stakeholder involvement, and clarity of presentation, and more variable for Rigour of development, applicability, and editorial independence.

**Results:**

We identified 32 core recommendations. For CHR-P, the most consistent components were specialized services or dedicated pathways, comprehensive multidisciplinary assessment, cognitive-behavioural therapy, family interventions, and a cautious approach to antipsychotic use. For FEP, the most consistently endorsed components were specialized multidisciplinary teams, assertive and continuous follow-up, family work, psychoeducation, structured pharmacological monitoring, and supported education and employment.

**Conclusion:**

These findings provide a guideline-derived framework for youth EIP service delivery and model development, while highlighting the need for more methodologically robust and implementation-oriented guidance across settings.

**Systematic review registration:**

https://osf.io/cek7u, identifier cek7u.

## 1 Introduction

Over the past three decades, youth mental health has emerged as a distinct field at the intersection of child and adult psychiatry, public health, and social policy. Mental disorders are now among the leading causes of disability and disease burden among people aged 10–24 years worldwide, with onset often occurring in adolescence and trajectories extending into adulthood, thereby generating substantial long-term individual and societal costs (1, 2). Recent global analyses and commissions have also highlighted a progressive deterioration in the mental health of emerging adults over the past two decades, driven by converging “megatrends” such as widening socioeconomic inequalities, employment precarity, climate anxiety, and the pervasive influence of poorly regulated digital environments (2, 3). In response, youth mental health scholars have called for a systematic reorientation of services toward earlier, developmentally informed, and staged models of care, supported by a coordinated research agenda focused on service delivery, implementation, and policy-relevant evidence (4).

Early intervention in psychosis (EIP) has been proposed as one of the clearest expressions of this reform agenda, linking developmentally sensitive detection strategies with phase-specific care for emerging psychosis (5–7). Psychotic disorders typically begin in late adolescence or early adulthood, and the first 3–5 years after onset are often regarded as a critical period during which clinical and functional trajectories may be particularly modifiable (8, 9). Across multiple cohorts, a longer duration of untreated psychosis has been consistently associated with more severe symptoms, poorer functional outcomes, greater service utilization, and higher societal costs (10–12). Within this framework, two clinical targets are especially relevant. The first is clinical high risk for psychosis (CHR-P), which refers to young people presenting with attenuated psychotic symptoms, brief intermittent psychotic symptoms, or a combination of functional decline and genetic vulnerability (13, 14). The second is first-episode psychosis (FEP), typically defined as the first occurrence of frank psychotic symptoms meeting diagnostic criteria for a psychotic disorder. For FEP, randomized trials and large observational studies suggest that specialized EIP services, compared with standard care, are associated with better symptomatic and functional outcomes, reduced hospitalization, and improved vocational participation (15–17). By contrast, the evidence base for preventive interventions in CHR-P remains more uncertain and contested, with meta-analyses suggesting modest and at times inconsistent advantages of specific pharmacological or psychosocial interventions over high-quality clinical monitoring and supportive care (18–20).

To translate these clinical and epidemiological advances into service reform, numerous clinical practice guidelines (CPGs) and quality standards (QSs) have been developed to inform EIP service design and care pathways for young people (21–23). CPGs are intended to support clinical and policy decision-making by synthesizing available evidence and expert consensus into graded recommendations, whereas QSs typically distill selected priorities into a smaller set of measurable indicators for commissioning and quality improvement (24, 25). However, these documents vary substantially in scope, target population, and methodological rigor. Some focus specifically on schizophrenia, others on broader psychotic disorders, and others still on transdiagnostic youth mental health populations; likewise, some address CHR-P, some FEP, and some both. They also differ in the extent to which they cover organizational dimensions such as service configuration, access and discharge criteria, and multidisciplinary team structure, as opposed to more strictly clinical aspects of assessment and treatment (21, 22, 26). This heterogeneity makes it difficult for clinicians, planners, and service leaders to design coherent, developmentally appropriate pathways of care across the psychosis-risk spectrum and to benchmark local services against international standards.

In addition to differences in scope and content, CPGs vary markedly in methodological quality. Systematic appraisals across medicine and mental health have shown that many guidelines lack transparent procedures for evidence synthesis, recommendation grading, stakeholder involvement, and management of conflicts of interest (27–29). The Appraisal of Guidelines for Research and Evaluation II (AGREE II) instrument provides a standardized framework for evaluating guideline quality across six domains: scope and purpose, stakeholder involvement, rigor of development, clarity of presentation, applicability, and editorial independence (30). In schizophrenia and related disorders, AGREE II-based reviews have reported substantial variability in quality, with particularly weak performance in rigor of development, applicability, and editorial independence, and only a minority of guidelines meeting predefined thresholds for high quality (31–33). Even when methodological standards are reasonably met, recommendations may remain heterogeneous or insufficiently specific, especially for complex service models such as EIP, where organizational features, access thresholds, and multidisciplinary team configuration are as consequential as individual clinical interventions (31). To date, however, no study has systematically applied AGREE II to guidelines specifically relevant to youth-focused EIP, nor synthesized their recommendations into a coherent set of core service components for CHR-P and FEP.

From a policy and systems perspective, this dual heterogeneity in methodological quality and substantive content raises two key questions. First, among the national and international CPGs and QSs addressing psychosis in adolescence and young adulthood, which documents meet acceptable methodological standards when systematically appraised using AGREE II? Second, across these documents, which recommendations emerge as core components of high-quality EIP services for individuals at CHR-P and for those with FEP? To our knowledge, no previous study has systematically evaluated the methodological quality of EIP-relevant guidelines for CHR-P and FEP while simultaneously mapping and synthesizing their recommendations on service configuration, assessment, and treatment. The present study addresses this gap through an AGREE II-based systematic review of international CPGs and QSs relevant to early intervention in psychosis in youth. Specifically, we aimed: (i) to appraise the methodological quality of these documents across all AGREE II domains; (ii) to extract and compare recommendations related to service organization, assessment strategies, and pharmacological and psychosocial interventions for CHR-P and FEP; and (iii) to identify a parsimonious set of guideline-derived core components that may inform the development and updating of EIP care pathways and youth mental health policy.

## 2 Materials and methods

### 2.1 Study design, registration, and search strategy

This study is a systematic review and critical appraisal of CPGs and QSs relevant to early intervention in psychosis. The review was designed and reported in accordance with the PRISMA 2020 statement (34). A protocol specifying the review questions, eligibility criteria, search approach, and planned analyses was developed *a priori* and registered on the Open Science Framework (OSF; cek7u) before manuscript submission (35). Because this review addressed clinical practice guidelines and quality standards rather than primary intervention studies, a full PICOS framework was not directly applicable. The review question was therefore operationalized around document type (CPGs/QSs), target population (CHR-P, FEP, or both, within youth-oriented or transition-age services), and recommendation domains (service organization, assessment, and treatment/intervention).

We searched for relevant CPGs and QSs published from 2005 to March 2025. Records were identified through MEDLINE (via PubMed), the Cochrane Library (CENTRAL), and Web of Science, and screened in two stages, consisting of title/abstract screening followed by full-text assessment of potentially eligible documents. Title/abstract screening and full-text assessment were conducted by two reviewers, with disagreements resolved through discussion; no automation tools were used. The electronic search combined terms related to psychosis risk states and early psychosis with terms related to guidelines, standards, recommendations, and service delivery. Because many relevant guidance documents are not consistently indexed in bibliographic databases, the database search was supplemented by targeted grey-literature searching, manual screening of reference lists from included documents and relevant reviews, and focused web-based retrieval from guideline repositories and websites of relevant governmental agencies, professional societies, non-profit organizations, and early psychosis networks. The complete PubMed search string used was: (“psychosis”[MeSH Terms] OR “psychotic disorders”[MeSH Terms] OR “first episode psychosis”[tiab] OR “first-episode psychosis”[tiab] OR “early psychosis”[tiab] OR “ultra-high risk”[tiab] OR “clinical high risk”[tiab] OR “CHR-P”[tiab] OR “at-risk mental state”[tiab] OR “attenuated psychosis syndrome”[tiab] OR “prodromal psychosis”[tiab]) AND (“practice guideline”[Publication Type] OR “guidelines as topic”[MeSH Terms] OR “clinical practice guideline*”[tiab] OR “quality standard*”[tiab] OR “recommendation*”[tiab] OR “consensus statement*”[tiab]) AND (“early intervention”[tiab] OR “youth mental health”[tiab] OR “young adult*”[tiab] OR “adolescent*”[MeSH Terms] OR “service delivery”[tiab] OR “specialized team*”[tiab] OR “assertive community”[tiab]). Conceptually equivalent source-adapted strategies were used for the Cochrane Library (CENTRAL) and Web of Science. The complete source-specific search strategies, browsing approaches, dates of consultation, and retrieval details for all databases and grey-literature sources are reported in **Supplementary Table 5**. No date filter beyond the 2005 lower limit was applied at the database level.

### 2.2 Eligibility criteria

We included documents that met all of the following criteria: (i) were presented as CPGs, QSs, or equivalent consensus- or standards-based guidance documents issued, commissioned, or formally endorsed by governmental agencies, professional societies, or non-profit organizations; (ii) were published from 2005 onward and were available in English or Italian; (iii) were relevant to early intervention in psychosis in adolescents or young adults, with specific recommendations addressing CHR-P, FEP, or both; and (iv) contained recommendations pertaining to at least one of the following domains: service organization (e.g., model of care, access, caseloads, duration of follow-up), assessment procedures (e.g., use of psychometric instruments, risk assessment, physical health monitoring), or pharmacological and psychosocial interventions. Because included documents used variable age thresholds and service definitions, the term “youth” is used in this review as an operational umbrella term referring to guidance addressing adolescence, young adulthood, or transition-age psychosis care rather than a single fixed age band. We excluded documents that: (i) focused exclusively on generic adult schizophrenia care without a distinct early-intervention, youth, CHR-P, or first-episode component; (ii) addressed only population-level screening or prevention without recommendations for the clinical assessment or management of CHR-P or FEP; (iii) were restricted to a single comorbidity or service setting (e.g., substance use or forensic services) without broader guidance on psychosis care pathways; or (iv) represented earlier versions that had been fully superseded by updated documents from the same issuing body. When multiple related documents were available (e.g., a full guideline and a derivative quality standard), each document was assessed separately if it contained distinct methodological information, recommendations, or implementation priorities.

### 2.3 Guideline appraisal

Methodological quality was appraised using AGREE II, which comprises 23 items grouped into six domains (Scope and purpose, Stakeholder involvement, Rigour of development, Clarity of presentation, Applicability, and Editorial independence), plus two overall assessment items (30). All items and both global ratings were scored on a 7-point Likert scale (1 = strongly disagree; 7 = strongly agree). Each appraised document was independently rated by two appraisers, and discrepancies were resolved through discussion; when consensus was not reached, the senior author acted as arbiter. Inter-rater agreement for AGREE II domain scores was assessed using intraclass correlation coefficients (two-way mixed effects model, absolute agreement). ICC values ranged from 0.82 to 0.91 across the six AGREE II domains, indicating good to excellent inter-rater reliability; domain-level ICC values and 95% confidence intervals are reported in **Supplementary Table 1A**. For each appraised document, standardized domain scores were calculated as percentages of the maximum possible score for that domain, in accordance with the AGREE II user manual (36). The overall assessment reflects the appraiser’s judgment regarding whether a document should be recommended for use in practice, but does not contribute to domain scores and was therefore considered descriptively. For descriptive purposes, documents with a mean overall assessment score below 5 were classified as “acceptable with modifications”, whereas those with a mean overall assessment score of 5 or higher were classified as “recommended for use”. Two included documents were judged not amenable to item-level AGREE II scoring because their format did not provide sufficient methodological detail for reliable domain-based appraisal. These documents were therefore excluded from formal AGREE II scoring and from AGREE II-based sensitivity analyses, but were retained in the recommendation synthesis because of their explicit EIP service focus and policy relevance.

### 2.4 Data extraction and synthesis of recommendations

We developed a structured data extraction form to collect, for each included document: (i) issuing organization, year of publication, country or region, and document type; (ii) scope and target population; (iii) methodological features, including the evidence-review approach, recommendation-grading system, stakeholder involvement, and updating procedures; and (iv) recommendations relevant to EIP. Recommendations were extracted and coded into three *a priori* domains: service configuration and organization, assessment, and treatment/intervention. Service-configuration items included, for example, model and setting of care, access and discharge criteria, duration and intensity of follow-up, multidisciplinary team composition, caseloads, and integration with other youth mental health services. Assessment items included the use of validated CHR-P instruments, diagnostic assessment of psychotic disorders, monitoring of symptoms, functioning and risk, and physical health assessment. Treatment/intervention items included antipsychotic and other pharmacological strategies, psychological therapies, family interventions, supported employment and education, and outreach and engagement activities. Where documents provided separate recommendation sets for CHR-P and FEP, these were extracted and coded independently.

Data extraction was performed independently in duplicate by two reviewers; discrepancies were resolved through discussion, and the senior author arbitrated when needed. Cohen’s kappa for inter-rater agreement was 0.83 (95% CI 0.79–0.87) at the title/abstract screening stage and 0.88 (95% CI 0.83–0.93) at the full-text eligibility assessment stage, indicating strong agreement at both stages. For cross-document synthesis, recommendations were considered at the document level rather than weighted by the number of statements within each document. Recommendation strength and directive wording were then harmonized across documents using a common three-level framework (mandatory/strong, recommended, optional/weak), while documents without an explicit grading system were classified separately as not graded. In the primary synthesis, endorsement frequency, harmonized recommendation strength, and AGREE II methodological quality were treated as analytically distinct dimensions. Frequency reflected how many documents endorsed a component at the document level; harmonized strength reflected the directive force of recommendations only where explicit grading or directive wording was available; and AGREE II scores were used to appraise document-level methodological quality rather than to weight recommendations in the primary classification. The operational crosswalk used for this harmonization is reported in **Supplementary Table 2**. Core components were defined *a priori* as recommendation categories endorsed by at least one third of the included documents relevant to the specific population and supported by at least one strong or mandatory recommendation across the contributing documents. This threshold was selected *a priori* as a pragmatic operational rule to identify recurrent components across heterogeneous guidance documents while avoiding an unduly permissive synthesis. It should therefore be interpreted as a rule for cross-document operational synthesis rather than as a formal consensus standard, although its logic is broadly consistent with previous systematic appraisals of guideline content and quality (36, 65). Frequencies were calculated separately for CHR-P and FEP because not all documents addressed both populations. To assess the robustness of the resulting framework, we also performed a *post hoc* sensitivity analysis using stricter operational criteria: first, raising the endorsement threshold from at least one third to at least one half of relevant documents; second, requiring support by at least two strong or mandatory recommendations instead of at least one; and third, examining the combined effect of both stricter criteria. As an additional quality-restricted sensitivity analysis, we also repeated the core-component classification after restricting the synthesis to formally appraised documents meeting a descriptive AGREE II quality indicator, defined as scores ≥60% in at least three domains including Rigour of development. Documents not amenable to item-level AGREE II scoring were not included in this quality-restricted analysis. Finally, we checked the internal consistency of the primary classification against the prespecified rule, given that some frequently endorsed components could be supported mainly by moderate, weak, or ungraded recommendations.

The completed PRISMA 2020 checklist is provided in **Supplementary Table 1**.

## 3 Results

### 3.1 Study selection

**Figure 1:**
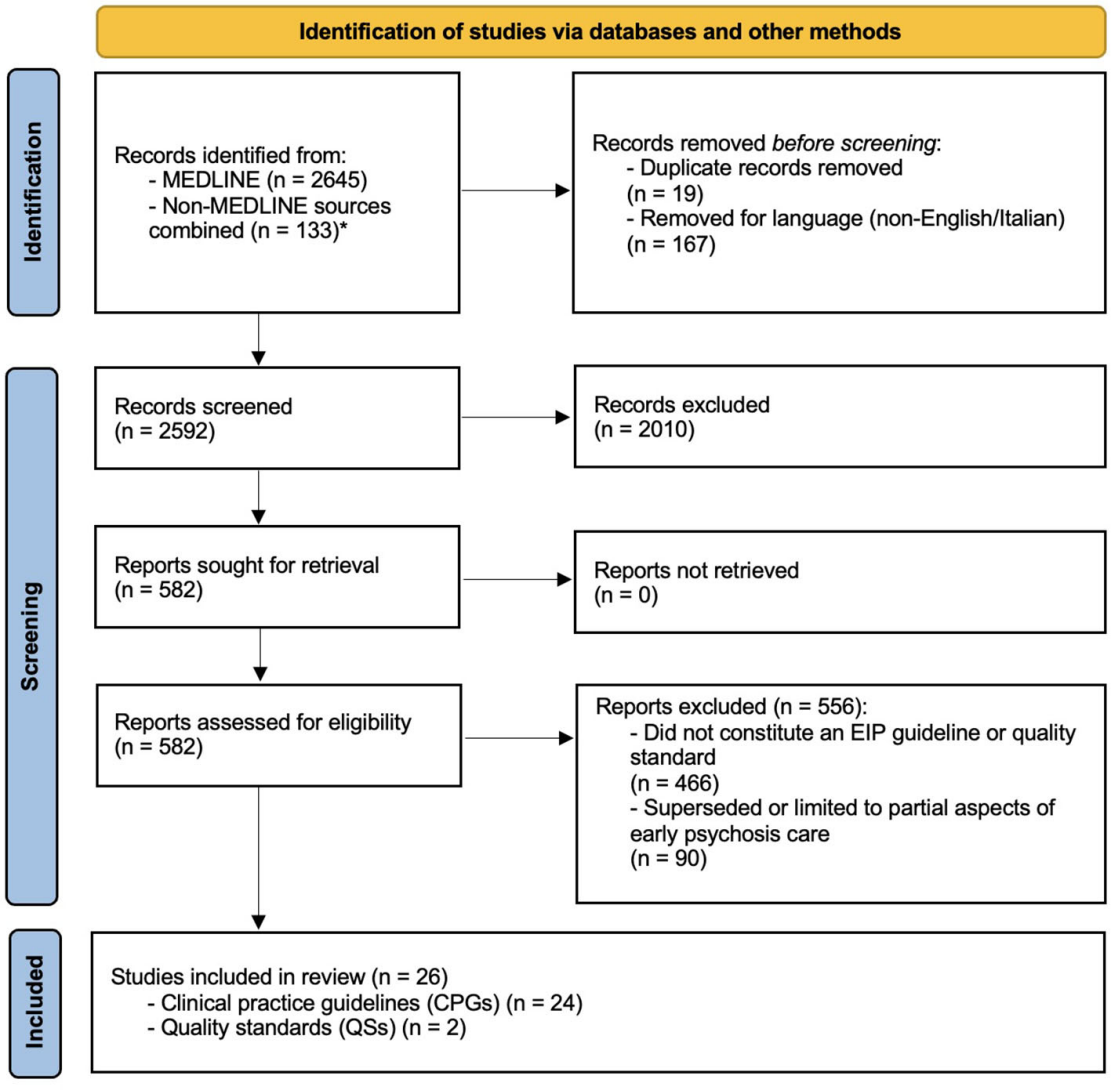
PRISMA 2020 flow diagram of the study selection process. The image summarizes the identification, screening, eligibility assessment, and final inclusion of documents in the review. A total of 26 documents were included, comprising 24 clinical practice guidelines and 2 quality standards. *Non-MEDLINE sources included CENTRAL, Web of Science, targeted grey-literature retrieval, and citation chasing.

The search and selection process is summarized in **Figure 1**. Overall, 2,778 records were identified, including 2,645 through MEDLINE (via PubMed) and 133 from non-MEDLINE sources combined. Before screening, 19 duplicate records and 167 records not available in English or Italian were removed, leaving 2,592 records for title and abstract screening. At this stage, 2,010 records were excluded as clearly not relevant to early intervention in psychosis. Full texts were sought for 582 reports, all of which were retrieved and assessed for eligibility. Of these, 556 were excluded: 466 because they did not constitute an EIP guideline or quality standard, and 90 because they had been superseded by updated versions from the same organization or were limited to partial aspects of early psychosis care. Ultimately, 26 documents met the inclusion criteria and were included in the review. The full list of excluded full-text reports with reasons for exclusion is provided in **Supplementary Table 6**.

### 3.2 Characteristics of included guidelines and quality standards

The final sample comprised 26 documents (21, 23, 26, 37–59), including 24 CPGs and 2 QSs, developed by governmental agencies, professional societies, and non-profit organizations (**Table 1**). Most were produced or commissioned by national or regional health authorities, with a smaller number originating from psychiatric associations or expert networks. The documents spanned four continents, with 13 from Europe, 9 from North America, 2 from Oceania, and 2 from Asia. Their scope and target populations were heterogeneous: some focused on schizophrenia-spectrum disorders across the lifespan, others on early psychosis or youth mental health more broadly, and only a minority were explicitly framed around dedicated EIP services. Almost all documents included recommendations for FEP, whereas 17 also provided specific guidance for CHR-P, most often embedded within broader schizophrenia or early psychosis guidance rather than published as standalone CHR-P CPGs. Age ranges were variably defined, usually covering adolescence and young adulthood (e.g., 12–35, 14–25, or 15–29 years), and several documents explicitly described youth-focused EIP service models. Accordingly, the present synthesis should be interpreted as applying to youth-oriented or transition-age EIP pathways rather than to a single uniformly age-defined population. The level of detail devoted to organizational features—such as catchment-area coverage, multidisciplinary team composition, assertive outreach, and interfaces with child and adolescent or primary care services—varied substantially across documents and was often less consistent than the coverage of symptom-focused assessment and treatment recommendations. Fourteen documents, including 13 CPGs and one set of EIP service standards, applied a formal system for grading the strength of recommendations and the underlying evidence, whereas the remainder relied on ungraded narrative or consensus-based statements.

**Table 1:** Characteristics of the 26 included clinical practice guidelines (CPGs) and quality standards (QSs).

Abbreviation/reference	Organization/institution	Title	Clinical focus*	Year
NICE CG 178 (21)	National Institute for Health and Care Excellence	Psychosis and Schizophrenia in Adults: Prevention and Management – CG178	CHR-P/FEP	2014
NICE QS 80 (23)	National Institute for Health and Care Excellence	Psychosis and Schizophrenia in Adults – Quality Standard No. 80	FEP	2015
RANZCP (26)	Royal Australian and New Zealand College of Psychiatrists	Clinical Practice Guidelines for the Management of Schizophrenia and Related Disorders	CHR-P/FEP	2016
CPA CHR-P (37)	Canadian Psychiatric Association	Canadian Treatment Guidelines for Individuals at Clinical High Risk of Psychosis	CHR-P	2017
APA (38)	American Psychiatric Association	Practice Guideline for the Treatment of Patients with Schizophrenia – 3rd ed.	FEP	2020
VA–DoD (39)	Department of Veterans Affairs; Department of Defense	VA/DoD Clinical Practice Guideline for Management of First-Episode Psychosis and Schizophrenia	CHR-P/FEP	2023
ANEP (40)	Asian Network for Early Psychosis	Early Psychosis Declaration for Asia by the Asian Network of Early Psychosis	FEP	2012
MHS British Columbia (41)	Ministry of Health Services, Province of British Columbia	Standards and Guidelines for Early Psychosis Intervention (EPI) Programs	CHR-P	2010
CAMH (42)	Centre for Addiction and Mental Health	First Episode Psychosis: An Information Guide (Revised ed.)	FEP	2015
ISS-SNLG (43)	Italian National Institute of Health; National Guidelines System	The Italian Guidelines for Early Intervention in Schizophrenia	CHR-P/FEP	2008
HSE (44)	Health Service Executive, Ireland	National Clinical Programme for Early Intervention in Psychosis – Model of Care	CHR-P/FEP	2019
IEPA (45)	International Early Psychosis Association	International Clinical Practice Guidelines for Early Psychosis	CHR-P/FEP	2005
IRIS (46)	Initiative to Reduce the Impact of Schizophrenia	Early Intervention in Psychosis Guidelines Update	CHR-P/FEP	2012
CPA FEP (47)	Canadian Psychiatric Association	Canadian Treatment Guidelines on Psychosocial Treatment of Schizophrenia in Children and Youth	FEP	2017
APP (48)	Asia-Pacific Psychiatry	Recommendations for the Optimal Care of Patients with Recent-Onset Psychosis in the Asia-Pacific Region	FEP	2016
MMHPI (49)	Meadows Mental Health Policy Institute	First Episode Psychosis Care: Best Practice Implications for the UTSW Psychosis Research Center	FEP	2016
NICE CG 155 (50)	National Institute for Health and Care Excellence	Psychosis and Schizophrenia in Children and Young People: Recognition and Management – CG155	CHR-P/FEP	2013
NCCMH–NICE (51)	NHS England; National Collaborating Centre for Mental Health	Implementing the Early Intervention in Psychosis Access and Waiting Time Standard	CHR-P/FEP	2023
MH Ontario (52)	Ministry of Health and Long-Term Care, Ontario	Early Psychosis Intervention Program Standards	CHR-P/FEP	2011
EASA (53)	Early Assessment and Support Alliance, Oregon	Practice Guidelines for Oregon EASA Programs	CHR-P/FEP	2014
Orygen (54)	Orygen National Centre of Excellence in Youth Mental Health	Australian Clinical Guidelines for Early Psychosis – 2nd ed.	CHR-P/FEP	2010
RCPsych (55)	Royal College of Psychiatrists	Standards for Early Intervention in Psychosis Services – 1st ed.	CHR-P/FEP	2021
EPA Intervention (56)	European Psychiatric Association	EPA Guidance on the Early Intervention in Clinical High Risk States of Psychosis	CHR-P	2015
EPA Detection (57)	European Psychiatric Association	EPA Guidance on the Early Detection of Clinical High Risk States of Psychosis	CHR-P	2015
SIGN (58)	Scottish Intercollegiate Guidelines Network	Management of Schizophrenia – National Clinical Guideline	FEP	2013
MHS Catalunya (59)	Ministry of Health, Generalitat de Catalunya	Clinical Practice Guideline for Schizophrenia and Incipient Psychotic Disorder	FEP	2009

*CHR-P, clinical high risk for psychosis; FEP, first-episode psychosis; CHR-P/FEP, document addresses both populations. Superscript numbers indicate bibliography reference positions in the final manuscript.

CPG, clinical practice guideline; QS, quality standard; EIP, early intervention in psychosis.

### 3.3 Methodological quality of guidelines

AGREE II domain scores for the 24 formally appraised documents are presented in **Table 2**. Two additional documents, ANEP (40) and RCPsych (55), were not amenable to item-level AGREE II scoring because their format did not provide sufficient methodological detail for reliable domain-based appraisal. They were therefore excluded from the formal AGREE II domain-score analysis, but retained in the recommendation synthesis because of their explicit EIP service focus and policy relevance. Their inclusion should therefore be interpreted as contributing to the descriptive mapping of recurrent service components rather than to the formal methodological appraisal of guideline quality. Methodological quality was moderate overall, with substantial variability across domains and across documents. The global AGREE II index, calculated as the average of the six standardised domain scores, ranged from 46.3% to 95.6%. It should be noted that the AGREE II manual does not prescribe a single aggregate score across domains; this index is used here for descriptive comparison only and should not be interpreted as a unidimensional quality metric. Scope and purpose was the best-performing domain (mean ± SD 88.6 ± 10.6), whereas Editorial independence showed the lowest mean score (63.1 ± 34.5). Stakeholder involvement exceeded the 60% threshold in 22 of 24 documents, whereas Clarity of presentation did so in 23 of 24, suggesting generally strong performance in the definition of target populations, intended users, and key recommendations.

**Table 2:** AGREE II domain scores and global index for the 24 formally appraised CPGs and QSs.

Abbreviation/reference	Scope and purpose (%)	Stakeholder involvement (%)	Rigour of development (%)	Clarity of presentation (%)	Applicability (%)	Editorial independence (%)	Global AGREE II index (%)	Domains ≥60%, n
NICE CG 178 (21)	100.0	94.4	97.2	96.3	90.3	94.4	95.4	6
NICE QS 80 (23)	100.0	94.4	82.6	94.4	73.6	86.1	88.5	6
RANZCP (26)	98.1	90.7	67.4	100.0	66.7	88.9	85.3	6
CPA CHR-P (37)	77.8	85.2	78.5	64.8	44.4	100.0	75.1	5
APA (38)	98.1	96.3	96.5	98.1	84.7	100.0	95.6	6
VA–DoD (39)	100.0	83.3	89.6	94.4	66.7	100.0	89.0	6
MHS British Columbia (41)	88.9	72.2	50.7	90.7	75.0	75.0	75.4	5
CAMH (42)	66.7	61.1	16.7	61.1	55.6	16.7	46.3	3
ISS-SNLG (43)	75.9	77.8	79.9	96.3	58.3	44.4	72.1	5
HSE (44)	98.1	88.9	47.2	77.8	93.1	5.6	68.5	4
IEPA (45)	83.3	74.1	34.0	92.6	37.5	16.7	56.4	3
IRIS (46)	96.3	81.5	21.5	90.7	77.8	11.1	63.1	4
CPA FEP (47)	64.8	79.6	67.4	60.3	45.8	91.7	68.3	5
APP (48)	94.4	77.8	72.2	88.9	58.3	66.7	76.4	5
MMHPI (49)	72.2	70.4	39.6	38.9	63.9	16.7	50.3	3
NICE CG 155 (50)	100.0	94.4	95.8	96.3	90.3	94.4	95.2	6
NCCMH–NICE (51)	88.9	85.2	70.1	87.0	81.9	66.7	80.0	6
MH Ontario (52)	88.9	61.1	27.8	72.2	37.5	63.9	58.6	4
EASA (53)	87.0	85.2	40.3	96.3	91.7	16.7	69.5	4
Orygen (54)	94.4	75.9	70.1	100.0	72.2	16.7	71.5	5
EPA Intervention (56)	87.0	50.0	77.8	70.4	44.4	83.3	68.8	4
EPA Detection (57)	87.0	50.0	77.8	70.4	44.4	83.3	68.8	4
SIGN (58)	94.4	92.6	78.5	88.9	73.6	83.3	85.2	6
MHS Catalunya (59)	83.3	83.3	78.5	87.0	68.1	91.7	82.0	6
**Mean ± SD**	**88.6 ± 10.6**	**79.4 ± 13.3**	**64.9 ± 24.2**	**83.9 ± 15.9**	**66.5 ± 17.7**	**63.1 ± 34.5**	**74.4 ± 13.7**	**—**

AGREE II domain scores are standardized percentages of the maximum achievable score per domain. The global AGREE II index is the mean of the six standardized domain scores (used for descriptive comparison; not prescribed by the AGREE II manual).

ANEP (ref 40) and RCPsych (ref 55) were excluded from formal AGREE II scoring owing to their document format; both were retained in the recommendation synthesis. ‘Domains ≥60%, n’, number of domains reaching or exceeding 60%. Bold values indicate the summary row reporting mean ± standard deviation across the 24 formally appraised documents.Bold values indicate the summary row reporting mean ± standard deviation across the 24 formally appraised documents.

Using this descriptive threshold, a substantial proportion of appraised documents met criteria for at least acceptable methodological quality, although important variability remained across domains and across documents. The AGREE II overall assessment ratings also indicated variability in the degree of confidence for direct use in practice: four documents received a mean overall score below 5 and were classified as “acceptable with modifications”, whereas the remaining 20 were judged “recommended for use”. This profile, with relatively high scores for scope and purpose, clarity of presentation, and stakeholder involvement, but more modest performance in rigour of development and editorial independence, is consistent with AGREE II-based appraisals of CPGs in other areas of medicine and mental health (27, 28, 33, 36). The identical AGREE II domain scores obtained by the EPA Guidance on Early Intervention (56) and the EPA Guidance on Early Detection (57) reflect their shared development process, authorship group, and methodological infrastructure within the same EPA task force publication series.

### 3.4 Core components of youth early intervention in psychosis services

Across the 26 included documents, **Table 3** summarizes 33 frequently endorsed service components across CHR-P and FEP. Under a strict application of the prespecified rule for core components, 32 of these met both criteria, namely endorsement by at least one third of the documents relevant to the specific population and support by at least one strong or mandatory recommendation. One CHR-P component, youth-friendly, low-stigma environments, met the frequency criterion (12/17 documents) but was not supported by any strong or mandatory recommendation and therefore did not meet the stricter definition in the internal consistency check. Overall, the synthesis showed substantial convergence across documents despite marked heterogeneity in scope, grading systems, and level of methodological detail. Seventeen documents provided recommendations relevant to CHR-P, whereas 22 addressed FEP; only 14 explicitly graded recommendation strength.

**Table 3:** Frequency and harmonized strength of the 33 recurrent service components across CPGs and QSs for CHR-P and FEP.

Pop.	Domain	Component	Documents endorsing, n/N (%)	Strong/mandatory, n	Moderate/recommended, n	Weak/optional, n	Not graded, n
CHR-P	Service configuration	Specialized multidisciplinary CHR-P services or dedicated pathways within EIP or youth mental health teams	16/17 (94.1)	4	2	3	7
CHR-P	Service configuration	Low-threshold and inclusive access routes	7/17 (41.2)	1	2	1	3
CHR-P	Service configuration	Integration with primary care and other mental health services	11/17 (64.7)	3	2	2	4
CHR-P	Service configuration	Proactive outreach and early detection activities	8/17 (47.1)	1	2	0	5
CHR-P	Service configuration	Youth-friendly, low-stigma environments†	12/17 (70.6)	0	2	2	8
CHR-P	Assessment	Comprehensive multidisciplinary biopsychosocial assessment	14/17 (82.4)	4	4	1	5
CHR-P	Assessment	Routine assessment of psychiatric and substance-use comorbidities	12/17 (70.6)	4	1	2	5
CHR-P	Assessment	Structured assessment of functioning and role performance	11/17 (64.7)	3	2	3	3
CHR-P	Assessment	Validated instruments/structured interviews for CHR-P identification and diagnostic formulation	10/17 (58.8)	3	2	3	2
CHR-P	Treatment	Cognitive-behavioural therapy (CBT)-based psychological interventions	12/17 (70.6)	5	2	1	4
CHR-P	Treatment	Family-focused interventions	12/17 (70.6)	5	2	1	4
CHR-P	Treatment	Psychoeducation	6/17 (35.3)	1	1	0	4
CHR-P	Treatment	Avoidance of routine antipsychotic use as first-line strategy	13/17 (76.5)	2	2	3	6
FEP	Service configuration	Specialized outpatient EIP teams	20/22 (90.9)	9	2	0	9
FEP	Service configuration	Timely and equitable access to care	15/22 (68.2)	3	3	3	6
FEP	Service configuration	Integration and coordination with child/adolescent, adult, emergency, and inpatient services	15/22 (68.2)	3	3	3	6
FEP	Service configuration	Assertive community treatment and/or intensive case management	16/22 (72.7)	4	3	2	7
FEP	Service configuration	Inpatient care when clinically indicated	9/22 (40.9)	2	1	2	4
FEP	Service configuration	Youth-friendly and non-stigmatizing settings	14/22 (63.6)	2	3	3	6
FEP	Service configuration	Structured policies for transition from youth to adult services	9/22 (40.9)	1	1	1	6
FEP	Assessment	Comprehensive multidisciplinary assessment at service entry	19/22 (86.4)	8	1	1	9
FEP	Assessment	Routine assessment of comorbid psychiatric and substance-use disorders	15/22 (68.2)	3	3	3	6
FEP	Assessment	Standardized assessment of functioning and disability	15/22 (68.2)	3	3	3	6
FEP	Assessment	Validated diagnostic or symptom-rating instruments for diagnosis and monitoring	16/22 (72.7)	4	3	2	7
FEP	Assessment	Regular structured review of physical health parameters	9/22 (40.9)	2	1	2	4
FEP	Treatment	Initiation of antipsychotic medication at low dose with gradual titration	15/22 (68.2)	6	0	2	7
FEP	Treatment	Systematic physical health and metabolic monitoring	21/22 (95.5)	8	0	2	11
FEP	Treatment	Timely consideration of clozapine in treatment-resistant cases	14/22 (63.6)	8	1	0	5
FEP	Treatment	CBT-informed psychological interventions for psychosis	20/22 (90.9)	10	1	0	9
FEP	Treatment	Structured family interventions	16/22 (72.7)	4	2	1	9
FEP	Treatment	Psychoeducation for service users and families	22/22 (100.0)	9	1	1	11
FEP	Treatment	Supported employment and education	18/22 (81.8)	7	1	2	8
FEP	Treatment	Cognitive remediation for persistent cognitive deficits	9/22 (40.9)	2	3	2	2

Under the prespecified core-component rule—endorsement by ≥1/3 of relevant documents plus ≥1 strong/mandatory recommendation—32 of the 33 recurrent components qualified as core components. Strength ratings were harmonized across grading systems (see **Supplementary Table 2**).

† Youth-friendly, low-stigma environments (CHR-P): met the frequency criterion (12/17, 70.6%) but had no strong or mandatory recommendation and therefore did not qualify as a core component under the strict prespecified rule; retained here as a descriptively endorsed component.

CHR-P, clinical high risk for psychosis; FEP, first-episode psychosis; CBT, cognitive-behavioural therapy; EIP, early intervention in psychosis; CPG, clinical practice guideline; QS, quality standard.

For CHR-P, the primary framework included 13 recurrent components, of which 12 satisfied the prespecified core-component rule in the strict consistency check. Organizational convergence centered on specialized multidisciplinary services or dedicated pathways within youth mental health or EIP teams (16/17), integration with primary care and other mental health services (11/17), low-threshold and inclusive access routes (7/17), proactive outreach and early detection activities (8/17), and youth-friendly, low-stigma environments (12/17), although the latter was supported only by moderate, weak, or ungraded statements. Assessment-related convergence was observed for comprehensive multidisciplinary biopsychosocial assessment (14/17), routine assessment of psychiatric and substance-use comorbidities (12/17), structured assessment of functioning and role performance (11/17), and the use of validated instruments or structured interviews for CHR-P identification and diagnostic formulation (10/17). Treatment-related convergence included CBT-based psychological interventions (12/17), family-focused interventions (12/17), psychoeducation (6/17), and avoidance of routine antipsychotic use as a first-line strategy in CHR-P (13/17), with antipsychotics generally reserved for more clearly defined clinical indications.

For FEP, 20 components met the prespecified core-component rule and the pattern of convergence was broader and more internally consistent than for CHR-P. Organizationally, most documents endorsed specialized outpatient EIP teams (20/22), timely and equitable access to care (15/22), integration and coordination with child and adolescent, adult, emergency, and inpatient services (15/22), assertive community treatment and/or intensive case management (16/22), inpatient care when clinically indicated (9/22), youth-friendly non-stigmatizing settings (14/22), and structured policies for transition from youth to adult services (9/22). Core assessment components included comprehensive multidisciplinary assessment at entry (19/22), routine assessment of comorbid psychiatric and substance-use disorders (15/22), standardized assessment of functioning and disability (15/22), validated diagnostic or symptom-rating instruments for diagnosis and monitoring (16/22), and regular structured review of physical health parameters (9/22). Treatment-related core components comprised low-dose initiation and gradual titration of antipsychotic medication with careful monitoring (15/22), systematic physical health and metabolic monitoring (21/22), timely consideration of clozapine in treatment-resistant cases (14/22), CBT-informed psychological interventions for psychosis (20/22), structured family interventions (16/22), psychoeducation for service users and families (22/22), supported employment and education (18/22), and cognitive remediation for persistent cognitive deficits (9/22). Taken together, these findings indicate broad cross-document agreement on the main organizational, assessment, and treatment elements of youth-oriented EIP care, with stronger and more stable convergence for FEP than for CHR-P.

Full document-level recommendation matrices for CHR-P and FEP are provided in **Supplementary Tables 3, 4**, respectively.

### 3.5 Patterns in the frequency and strength of recommendations

**Table 3** summarizes, for each recurrent component, both the frequency of endorsement across documents and, where available, the harmonized strength of recommendation. Overall, 14 of the 26 included documents applied an explicit grading system, whereas the remainder relied on narrative, consensus-based, or standards-oriented formulations without a formal strength taxonomy. Even among graded documents, grading systems differed substantially and required harmonization into a common three-level framework. Methodological quality, however, was appraised separately using AGREE II and should not be conflated with either endorsement frequency or directive strength. Accordingly, a component could be frequently endorsed across documents, strongly worded in a subset of them, or derived partly from documents of higher or lower methodological quality, and these dimensions did not necessarily coincide. In general, broad organizational principles, comprehensive assessment processes, and some core treatment elements were more likely to attract at least one strong or mandatory endorsement, whereas more specific or implementation-sensitive service features were more often supported by moderate, weak, or ungraded statements.

For CHR-P, the main pattern was a partial dissociation between how often a component was endorsed and how strongly it was graded. Specialized multidisciplinary CHR-P services or dedicated pathways, comprehensive multidisciplinary assessment, routine assessment of psychiatric and substance-use comorbidities, structured assessment of functioning, and the use of validated instruments or structured interviews were all recurrently endorsed, but their grading remained variable across documents. By contrast, avoidance of routine antipsychotic use as a first-line strategy showed a more consistent pattern of strong or mandatory support where grading was reported. CBT-based and family-focused interventions were also widely endorsed, although their strength ratings ranged from strong to weak across sources. The clearest example of a frequency-strength mismatch was youth-friendly, low-stigma environments, which was endorsed by 12 of 17 CHR-P-relevant documents but was not supported by any strong or mandatory recommendation, explaining why it was retained in the broader descriptive framework but did not meet the stricter prespecified rule in the consistency check.

Among FEP recommendations, the pattern was more internally consistent. Several components combined high endorsement frequency with at least some strong or mandatory support across documents, including specialized outpatient EIP teams, assertive community treatment or intensive case management, comprehensive multidisciplinary assessment, validated diagnostic or symptom-rating instruments, systematic physical health and metabolic monitoring, CBT-informed psychological interventions, psychoeducation, and supported employment and education. At the same time, not all frequently endorsed psychosocial or service-organizational components were uniformly strongly graded. Structured family interventions, youth-friendly settings, and supported employment and education showed more mixed strength profiles, while inpatient care when clinically indicated, structured transition policies, regular structured review of physical health parameters, and cognitive remediation met the primary threshold but remained closer to the lower boundary of endorsement frequency. Overall, the FEP literature displayed stronger convergence not only in what was recommended, but also in how often those recommendations were framed in directive terms.

The sensitivity analysis supported the overall stability of the framework while showing that some components were more threshold-sensitive than others. When the endorsement threshold was raised from at least one third to at least one half of relevant documents, 25 of the 32 strictly defined core components were retained, comprising 9 CHR-P and 16 FEP components. When the criterion was instead tightened to require at least two strong or mandatory recommendations, 28 of 32 components were retained, comprising 9 CHR-P and 19 FEP components. Applying both stricter criteria jointly again yielded 25 retained components. The components most sensitive to these stricter thresholds were, in CHR-P, low-threshold access routes, proactive outreach and early detection activities, and psychoeducation, and, in FEP, inpatient care when clinically indicated, structured transition policies, regular structured review of physical health parameters, and cognitive remediation. Taken together, these findings indicate that the proposed framework is reasonably robust at the level of its main organizational, assessment, and treatment architecture, while also identifying a smaller group of more variably endorsed components that may be better interpreted as important but less stable peripheral elements.

As an additional quality-restricted sensitivity analysis, we repeated the core-component classification after limiting the synthesis to formally appraised documents meeting the descriptive AGREE II quality indicator used in this review (≥60% in at least three domains including Rigour of development). Sixteen of the 24 formally appraised documents met this criterion, whereas documents not amenable to item-level AGREE II scoring were not included in this restricted analysis. Within this subset, 9 of the 12 strictly defined CHR-P core components and 19 of the 20 FEP core components remained above threshold. The CHR-P components no longer retained under this restriction were low-threshold and inclusive access routes, proactive outreach and early detection activities, and psychoeducation, whereas for FEP the only component no longer retained was structured policies for transition from youth to adult services. Overall, this analysis suggested that the core framework was largely robust to exclusion of lower-quality guidance, while a small number of more implementation-sensitive components appeared more quality-sensitive. This also indicates that the main architecture of the framework was not driven by inclusion of the two documents that could not be formally appraised with AGREE II.

## 4 Discussion

### 4.1 Summary of main findings

This systematic review and AGREE II-based appraisal of 26 international documents indicates that CPGs and QSs relevant to youth-oriented early intervention in psychosis are now sufficiently developed to support a cross-jurisdictional synthesis of core service components, although not a fully standardized model of care. Overall methodological quality was moderate, with the strongest performance in scope and purpose and clarity of presentation, and more variable performance in rigour of development, applicability, and editorial independence. This pattern is consistent with previous comparisons of schizophrenia and mental health guidelines, which have similarly found that recommendations are often clearer than the methods and implementation tools underpinning them. The weaker performance in applicability is particularly important in the EIP field, where organizational feasibility, transition pathways, and service integration are central determinants of whether guidance can actually be translated into routine care (24, 27–33).

Across these heterogeneous documents, we identified 32 core recommendations that together delineate a recognizable model of youth-oriented EIP care. For CHR-P, convergence was strongest around specialized or clearly delineated pathways, comprehensive multidisciplinary assessment using validated instruments, and a cautious, stepped-care approach privileging psychological and family-based interventions while avoiding routine antipsychotic use. For FEP, convergence was stronger and more internally consistent, centering on specialized multidisciplinary teams, assertive and community-based follow-up, structured pharmacological treatment and monitoring, psychoeducation, family work, and support for educational and vocational recovery. These findings are broadly aligned with earlier work on “good practice” in EIP and with the wider evidence base suggesting that early psychosis services are most effective when delivered as organized multicomponent packages rather than as isolated interventions (7, 16, 19, 22, 31, 43, 47, 50, 60, 61).

At the same time, our results indicate that not all core components are supported by the same degree of evidentiary certainty or methodological transparency. In CHR-P, convergence was stronger around service ethos, access pathways, and clinical prudence than around the efficacy of specific preventive interventions, which is consistent with the continuing uncertainty of the preventive treatment literature and with more recent synoptic analyses of CHR-P guidelines. Within this context, outreach, low-threshold access, and youth-friendly, low-stigma entry pathways may be important not only for earlier case identification, but also for reducing help-seeking delay and limiting the pathologizing effects of referral routes embedded in generic mental health services. In FEP, by contrast, both the evidence base and the guideline-derived model appeared more stable, especially for specialized team-based care, continuity of follow-up, and structured pharmacological and psychosocial treatment. Taken together, these findings suggest that the framework identified here should be interpreted as a structured synthesis of current international guideline practice rather than as a fixed or universally transferable standard of care, while also highlighting clear priorities for future guideline development, implementation work, and empirical refinement of youth EIP models (18, 19, 31, 50, 51, 60–64). Accordingly, the framework should be read as an operational synthesis of recurrent guideline content rather than as a definitive consensus standard or a direct proxy for comparative effectiveness. In particular, recurrent endorsement across documents should not be interpreted as equivalent either to uniformly strong recommendation wording or to uniformly high methodological quality of the contributing guidance.

### 4.2 Methodological quality of early psychosis guidelines

Overall, the methodological quality of the included documents was moderate rather than uniformly high when appraised with AGREE II. Most scored well on scope and purpose and clarity of presentation, suggesting that target populations, clinical aims, and key recommendations were generally well defined and clearly worded. This profile is broadly consistent with previous AGREE II-based appraisals in mental health and general medicine, which have repeatedly shown that guideline groups are often more successful in articulating recommendations than in documenting the evidentiary and implementation processes that support them (27–29, 31–33, 36).

By contrast, several domains that are crucial for the trustworthiness, usability, and real-world uptake of guidelines were more variable and often weaker. Stakeholder involvement was inconsistently reported, with relatively few documents clearly describing meaningful participation of young people, families, or peer-support perspectives in development processes, despite the centrality of these actors in youth-oriented and recovery-focused models of care. This is not unique to early psychosis guidance: other appraisals of mental health guidelines have similarly found that family and service-user perspectives are often insufficiently integrated or poorly reported, even when family involvement is later emphasized in the recommendations themselves (31, 65). Applicability was also commonly underdeveloped. Implementation tools, resource implications, cost considerations, and audit criteria were frequently absent or only briefly mentioned, even where recommendations implied substantial service redesign, workforce development, or investment in specialized teams. This limitation is especially relevant in EIP, where successful uptake depends not only on clinical consensus but also on referral pathways, staffing models, inter-service coordination, and sustained organizational support (32, 64, 66). Editorial independence and conflict-of-interest management were likewise unevenly reported, with some documents providing transparent declarations and funding statements and others offering minimal information, a pattern already described in broader guideline-quality reviews (27, 28, 33).

These findings have several implications. First, they suggest that even when recommendations are conceptually strong and broadly aligned with the evidence base, guideline documents may still provide insufficient support for implementation, particularly in under-resourced systems or in settings where EIP remains only partially established (32, 64, 66). Second, the relative neglect of stakeholder involvement is somewhat at odds with the youth-oriented, recovery-focused ethos that many documents espouse at the level of content, and may weaken both legitimacy and local acceptability of recommendations (31, 65). Third, the AGREE II profile reinforces the point that strong performance in selected domains does not automatically ensure that guidelines are up to date, unbiased, or readily adaptable to local service ecologies (30, 36). For this reason, the combined approach adopted in the present review—methodological appraisal alongside structured synthesis of recommendation content—appears particularly appropriate in early psychosis, where the practical value of guidance depends as much on implementability and contextual fit as on formal methodological quality alone.

### 4.3 Core components of EIP services

The 32 core recommendations identified in this review converge on a model of early intervention that is both phase-specific and system-level. Rather than being limited to isolated clinical interventions, the core components describe how services should be configured, how care should be organized along the pathway from initial help-seeking to longer-term recovery, and how pharmacological, psychological, social, and vocational modalities should be combined within a coherent service package (7, 16, 22, 31, 43, 47, 50, 60, 61). This degree of convergence is notable given the heterogeneity of health systems, funding arrangements, and professional cultures represented in the included documents. Taken together, these components depict EIP as a youth-focused, multidisciplinary, assertive, and recovery-oriented model designed not only to reduce treatment delay and attenuate the harms of emerging psychosis, but also to preserve social participation and functional development during a critical developmental period (5–7, 15–17, 50, 60).

A first key observation is that many core components are organizational rather than narrowly clinical. Specialized EIP teams, clear catchment-area responsibility, active outreach, defined pathways of entry and discharge, and intensive case management were among the most consistently endorsed elements, particularly for FEP. This is consistent with the broader EIP literature, which increasingly suggests that outcomes are linked not simply to the presence of specific treatments, but to the way these treatments are embedded within service structures capable of sustaining engagement, continuity, and timely responsiveness (16, 17, 31, 47, 50, 60, 61). In this respect, organizational design is not merely a contextual backdrop to good care; it is part of the intervention itself. The recurrent emphasis on shared care with primary care providers, coordination with emergency and inpatient services, and structured transition planning also reinforces the view of EIP as a service “hub” within a larger network of youth mental health and general health provision. This interpretation is further supported by implementation research showing that funding, staffing, inter-service collaboration, and communication pathways are among the most consistent determinants of whether EIP models can be sustained in routine practice (64, 66).

A second observation is that the core components for CHR-P and FEP reveal both continuity and asymmetry across the psychosis-risk spectrum. On the one hand, both phases are framed as requiring multidisciplinary assessment, structured formulation of symptoms and risk, attention to functioning and comorbidity, and a strong psychosocial backbone including family work and developmentally appropriate support (18–20, 31, 50, 62). On the other hand, pharmacological treatment occupies a very different place in the two phases. For FEP, antipsychotic treatment, physical-health monitoring, and timely clozapine use in treatment-resistant cases are central parts of the model; for CHR-P, by contrast, medication is largely framed in restrictive or precautionary terms, with guidelines converging on avoidance of routine antipsychotic use and prioritization of psychosocial care (18–20, 31, 50, 62, 63). This asymmetry likely reflects both the stronger evidence base for phase-specific treatment in FEP and the persisting uncertainty surrounding preventive interventions in CHR-P, where contemporary meta-analytic work continues to show limited and inconsistent superiority of specific interventions over high-quality monitoring and supportive care (19, 20, 62, 63).

A third observation is that the widespread inclusion of psychosocial, family, physical-health, and vocational interventions as core components indicates a substantive normative orientation in the guideline literature. EIP is not presented as a narrow symptom-management strategy, but as a multidimensional service model that routinely incorporates psychoeducation, family involvement, support for education and employment, and interventions targeting cognition, physical health, and social recovery (16, 17, 43, 47, 50, 60, 61). Recent component-level analyses are broadly consistent with this emphasis, suggesting that case management and psychological interventions contribute importantly to sustained benefit within early psychosis services, while national cohort data indicate that smaller case-loads, clozapine use for eligible service users, and physical-health interventions may be particularly consequential for relapse and mortality outcomes (60, 61). At the same time, the inclusion of these components as “core” exposes a persistent tension between what guidelines define as constitutive of good care and what many systems are currently able to fund, staff, and implement in practice, especially when vocational or recovery-oriented elements remain less protected than pharmacological or crisis-response functions (64).

Finally, the core-component framework emerging from this review has value beyond descriptive synthesis. For policy-makers and service leaders, it offers a concise, cross-nationally derived reference model that may inform service design, benchmarking, evaluation, and, where relevant, accreditation processes (24, 25, 50, 64). For researchers, it delineates clusters of components—organizational, assessment-related, pharmacological, psychosocial, and vocational—that can be tested in combination, rather than as isolated techniques, to clarify which configurations are most important for which outcomes and in which contexts (60, 61). At the same time, the variability in recommendation grading and the uneven treatment of implementation issues indicate that this framework should not be used as a rigid checklist. It is better understood as a structured reference point that requires contextual adaptation to local resources, service histories, governance arrangements, and the priorities of young people and families (30, 64, 66).

### 4.4 Implications for policy, practice, and research

Taken together, these findings suggest that the value of EIP guidelines lies not only in summarizing available evidence, but in making explicit what a youth-oriented early psychosis service is expected to be, organize, and deliver. At the policy level, the convergence of recommendations across diverse jurisdictions supports the use of EIP guidance as a basis for defining minimum standards for youth psychosis care, while the persistent weaknesses in applicability and implementation detail make equally clear that publication alone is not enough to change practice (24, 25, 30, 64, 66). In this sense, the framework identified in the present review may help policy-makers, commissioners, and service leaders move beyond generic invocations of “early intervention” toward more concrete specifications of service configuration, access pathways, transition arrangements, and integrated psychosocial and vocational provision. This is especially relevant in systems where early psychosis care is still underdeveloped or remains embedded within generic community structures, because what is needed is not rhetorical endorsement of EIP, but an operational model that can be progressively implemented, resourced, and evaluated (24, 25, 50, 64).

For clinical services, the implications are equally clear. EIP cannot be reduced to the presence of a dedicated team label or to the earlier prescription of antipsychotic medication. The guidelines reviewed here consistently frame EIP as a multicomponent model in which organizational architecture and clinical content are inseparable: multidisciplinary assessment, continuity of care, family involvement, psychoeducation, physical-health monitoring, and educational or vocational support are not optional accessories, but part of the intervention itself (16, 17, 43, 47, 50, 60, 61). At the same time, the variable strength and transparency of recommendations, together with the limited attention to implementation tools, argues against any rigid transplantation of guideline models from one setting to another. Adaptation remains necessary, but it should be principled rather than opportunistic, and should be guided by local resources, service histories, and the priorities of young people and families rather than by institutional inertia alone (30, 64, 66).

The implications are particularly sharp for CHR-P care. The consistent recommendation to avoid routine antipsychotic treatment while privileging psychosocial and family-based interventions makes clear that CHR-P pathways should not be treated as merely attenuated versions of FEP programs, but as a distinct form of preventive and uncertainty-sensitive care (18–20, 31, 50, 62, 63). This has practical consequences. Where CHR-P and FEP are managed within the same team, services need explicit thresholds for escalation, robust supervision, and genuinely shared decision-making processes, both to avoid premature pharmacological drift and to prevent delays when a transition to frank psychosis occurs. In this area, guideline quality matters directly because the clinical terrain is marked not only by vulnerability and risk, but also by prognostic ambiguity and the possibility of overtreatment.

From a research perspective, the framework proposed here offers a pragmatic architecture for moving beyond the recurrent problem that EIP is evaluated as a complex package whose active ingredients remain only partially disentangled. Grouping recommendations into organizational, assessment-related, pharmacological, psychosocial, and vocational clusters may help structure comparative studies, implementation trials, and natural experiments capable of testing which combinations of components matter most for which outcomes and in which contexts (60, 61, 64). At the same time, the present findings also indicate that future guideline development should improve not only in evidentiary rigor, but in stakeholder involvement, transparency of grading, reporting of conflicts of interest, and provision of implementation tools that make recommendations usable in real systems rather than merely defensible on paper (27–33, 65, 66).

A final interpretive implication concerns the institutional role of guidelines in contexts marked by uncertainty, risk, and uneven service capacity. In youth psychosis care, guidelines do not merely summarize evidence; they also help shape what may be regarded as timely, proportionate, and accountable care. From this perspective, methodological weakness, limited implementability, or insufficient contextual specificity may have consequences that extend beyond technical quality, because they may leave clinicians and services with less support for making difficult decisions consistently and transparently. This consideration should not be read as a direct empirical finding of the present review, but rather as a policy-relevant implication of the observed variability in guideline quality, grading transparency, and implementation detail. This interpretation is compatible with broader literature showing that perceived medico-legal exposure and institutional uncertainty can influence clinical decision-making, including in psychiatry and, specifically, in Italian psychiatric practice (67–69). Strengthening the methodological quality and practical usability of EIP guidance may therefore be relevant not only to service design, but also to supporting more consistent and accountable care.

### 4.5 Strengths and limitations

This review has several strengths. To our knowledge, it is the first to combine a systematic search for international early psychosis guidance documents with a formal AGREE II appraisal and a structured synthesis of core service components for both CHR-P and FEP care. The review adopted explicit eligibility criteria, duplicate screening and data extraction procedures, and a transparent framework for both methodological appraisal and cross-document synthesis. In addition, the decision to combine bibliographic searching with targeted retrieval of policy-oriented and grey-literature documents increased the likelihood of capturing guidance materials that are highly relevant to service design but are not always well represented in conventional databases. Finally, by moving beyond a purely descriptive summary of recommendations, the core-component analysis provides a more operational account of what contemporary documents recurrently define as constitutive of EIP care.

At the same time, several limitations should be acknowledged. First, the review was restricted to documents available in English or Italian, which may have led to underrepresentation of guidance developed in other linguistic and health-system contexts and may limit the global generalizability of the findings. Second, although MEDLINE (via PubMed), the Cochrane Library (CENTRAL), and Web of Science were searched, access to EMBASE and PsycINFO was not available, which may have led to under retrieval of conference proceedings, grey literature, and non-English documents indexed in those databases. Retrieval relied on a combination of bibliographic and targeted source searching; although this improved capture of jurisdiction-specific materials, reproducibility is reduced compared with a fully database-driven strategy. Third, despite efforts to identify grey literature, some local, unpublished, or non-indexed documents may still have been missed, particularly in settings where EIP services have developed without strong national coordination. Fourth, AGREE II evaluates the methodological quality and reporting of guideline development rather than the intrinsic validity or clinical superiority of individual recommendations. Its ratings, although generated through duplicate appraisal and adjudication, retain an element of judgment and should therefore be interpreted as indicators of transparency and methodological robustness rather than as definitive rankings of clinical merit. Fifth, two included documents were not amenable to item-level AGREE II scoring because their format did not provide sufficient methodological detail for reliable domain-based appraisal. They were therefore excluded from the formal AGREE II analyses, although they were retained in the descriptive recommendation synthesis because of their direct relevance to EIP service design. Reassuringly, the quality-restricted sensitivity analysis suggested that the main framework was not materially dependent on their inclusion. Sixth, the synthesis of recommendation strength was constrained by substantial heterogeneity in grading systems across documents. For the same reason, frequency of endorsement, harmonized directive strength, and methodological quality should be interpreted as complementary rather than interchangeable indicators. Harmonization into three broad categories was necessary for comparison, but inevitably simplified differences in how guideline groups weighed evidence, consensus, feasibility, and patient preferences. Seventh, the definition of “core” components was based on a pragmatic frequency threshold combined with the presence of at least one strong or mandatory recommendation. This approach was useful for identifying recurrent elements across heterogeneous documents, but it does not constitute a direct estimate of effectiveness or cost-effectiveness, nor does it imply that components falling below threshold are unimportant in specific contexts. Reassuringly, the quality-restricted sensitivity analysis showed broad stability of the framework, although a small number of more implementation-sensitive components appeared more sensitive to exclusion of lower-quality guidance. Eighth, the review focused on what documents recommend rather than on what services actually deliver. As a result, the identified framework should not be conflated with real-world implementation, which is likely to remain uneven, especially for resource-intensive psychosocial, family-based, and vocational elements. Finally, several included documents were developed years ago and may not fully reflect more recent developments in youth mental health service design, digital supports, trauma-informed approaches, or emerging evidence in preventive psychiatry. The framework proposed here should therefore be understood as a structured, context-sensitive synthesis of current guideline practice rather than as a fixed or exhaustive standard of care.

## 5 Conclusion

This review suggests that early intervention in psychosis is no longer conceptualized internationally as a vague aspiration or a generic call for earlier treatment, but as a recognizable model of youth mental health care with recurrent organizational, clinical, psychosocial, and vocational components. Across heterogeneous CPGs and QSs, we identified a sufficiently stable cross-national pattern to support a guideline-derived framework for CHR-P and FEP services, while also showing that methodological quality, recommendation grading, and implementation guidance remain uneven. The resulting framework should therefore not be read as a fixed or universally transferable standard of care, but as a structured map of what contemporary guideline practice most consistently regards as constitutive of EIP.

The main value of this synthesis lies in making explicit that good EIP care is not reducible to isolated clinical interventions. It depends on how services are built: whether access is timely, whether multidisciplinary assessment is available, whether follow-up is assertive and developmentally appropriate, whether family, psychosocial, physical-health, educational, and vocational interventions are actually integrated into routine care, and whether preventive decisions are supported by clear and proportionate pathways across the psychosis-risk spectrum. In this sense, the present findings are relevant not only for clinicians, but for policy-makers, commissioners, and service leaders, because they clarify what must be in place for “early intervention” to be more than a label.

At the same time, this review also points to a broader challenge. In youth psychosis, guidelines do not merely summarize evidence; they also help shape what may be regarded as timely, proportionate, and accountable care in contexts marked by uncertainty, risk, and unequal resources. Their quality therefore matters not only methodologically, but also ethically and institutionally. Stronger EIP guidelines should not merely provide clearer recommendations: they should also become more transparent, more implementable, more attentive to stakeholder perspectives, and more useful in real-world systems where prevention, recovery, and responsibility must be held together. The next task is not only to refine the evidence base for individual components, but also to develop guidance that can effectively support sustainable, context-sensitive, and accountable youth mental health services. It is worth noting that the present review, though conducted with an explicit Italian-language inclusion criterion, identified only one previously existing Italian document (43). More recent Italian institutional guidance also suggests that EIP guidance development remains active at sub-national and regional level in Italy (70); however, documents identified after completion of the systematic search fall outside the present synthesis. Guidance updates published after the March 2025 search cut-off are noted for completeness but were not included in the present synthesis.

## Data Availability

The original contributions presented in the study are included in the article/supplementary material. Further inquiries can be directed to the corresponding author.

## References

[1] Patel V Flisher AJ Hetrick S McGorry P . Mental health of young people: a global public-health challenge. Lancet. (2007) 369:1302–13. doi: 10.1016/S0140-6736(07)60368-7. PMID: 17434406

[2] McGorry PD Mei C Dalal N Alvarez-Jimenez M Blakemore SJ Browne V . The Lancet Psychiatry Commission on youth mental health. Lancet Psychiatry. (2024) 11:731–74. doi: 10.1016/S2215-0366(24)00163-9. PMID: 39147461

[3] Rose-Clarke K Bitta M Evans-Lacko S Jokinen T Nyongesa MK Patalay P . Centring youth mental health discourse on low-income and middle-income countries. Lancet Psychiatry. (2024) 11:671–2. doi: 10.1016/S2215-0366(24)00211-6. PMID: 39147454

[4] Mei C Fitzsimons J Allen N Alvarez-Jimenez M Amminger GP Browne V . Global research priorities for youth mental health. Early Interv Psychiatry. (2020) 14:3–13. doi: 10.1111/eip.12878. PMID: 31960595

[5] McGorry PD Killackey E Yung A . Early intervention in psychosis: concepts, evidence and future directions. World Psychiatry. (2008) 7:148–56. doi: 10.1002/j.2051-5545.2008.tb00182.x. PMID: 18836582 PMC2559918

[6] Birchwood M Lester H McCarthy L Jones PB Fowler D Amos T . The UK National Evaluation of the Development and Impact of Early Intervention Services (the NEDEN study): access, engagement and outcomes. Br J Psychiatry. (2014) 205:440–8. doi: 10.1192/bjp.bp.113.128660. PMID: 23347742

[7] Correll CU Galling B Pawar A Krivko A Bonetto C Ruggeri M . Comparison of early intervention services vs treatment as usual for early-phase psychosis: a systematic review and meta-analysis. JAMA Psychiatry. (2018) 75:555–65. doi: 10.1001/jamapsychiatry.2018.0623. PMID: 29800949 PMC6137532

[8] Birchwood M Todd P Jackson C . Early intervention in psychosis: the critical period hypothesis. Br J Psychiatry Suppl. (1998) 172:53–9. doi: 10.1192/S0007125000297663 9764127

[9] Norman RM Lewis SW Marshall M . Duration of untreated psychosis and its relationship to clinical outcome. Br J Psychiatry Suppl. (2005) 187:s19–23. doi: 10.1192/bjp.187.48.s19. PMID: 16055802

[10] Penttilä M Jääskeläinen E Hirvonen N Isohanni M Miettunen J . Duration of untreated psychosis as a predictor of long-term outcome in schizophrenia: systematic review and meta-analysis. Br J Psychiatry. (2014) 205:88–94. doi: 10.1192/bjp.bp.113.127753. PMID: 25252316

[11] Marshall M Lewis S Lockwood A Drake R Jones P Croudace T . Association between duration of untreated psychosis and outcome in cohorts of first-episode patients: a systematic review. Arch Gen Psychiatry. (2005) 62:975–83. doi: 10.1001/archpsyc.62.9.975. PMID: 16143729

[12] Knapp M Andrew A McDaid D Iemmi V McCrone P Park AL . Investing in recovery: making the business case for effective interventions for people with schizophrenia and psychosis. London: Rethink Mental Illness (2014).

[13] Fusar-Poli P Borgwardt S Bechdolf A Addington J Riecher-Rössler A Schultze-Lutter F . The psychosis high-risk state: a comprehensive state-of-the-art review. JAMA Psychiatry. (2013) 70:107–20. doi: 10.1001/jamapsychiatry.2013.269. PMID: 23165428 PMC4356506

[14] Fusar-Poli P Bonoldi I Yung AR Borgwardt S Kempton MJ Valmaggia L . Predicting psychosis: meta-analysis of transition outcomes in individuals at high clinical risk. Arch Gen Psychiatry. (2012) 69:220–9. doi: 10.1001/archgenpsychiatry.2011.1472. PMID: 22393215

[15] Craig TKJ Garety P Power P Rahaman N Colbert S Fornells-Ambrojo M . The Lambeth Early Onset (LEO) Team: randomised controlled trial of the effectiveness of specialised care for early psychosis. BMJ. (2004) 329:1067. doi: 10.1136/bmj.38246.594873.7C. PMID: 15485934 PMC526115

[16] Kane JM Robinson DG Schooler NR Mueser KT Penn DL Rosenheck RA . Comprehensive versus usual community care for first-episode psychosis: 2-year outcomes from the NIMH RAISE-ETP study. Am J Psychiatry. (2016) 173:362–72. doi: 10.1176/appi.ajp.2015.15050632. PMID: 26481174 PMC4981493

[17] Secher RG Hjorthøj CR Austin SF Thorup A Jeppesen P Mors O . Ten-year follow-up of the OPUS specialized early intervention trial for patients with a first episode of psychosis. Schizophr Bull. (2014) 41:617–26. doi: 10.1093/schbul/sbu155. PMID: 25381449 PMC4393691

[18] Fusar-Poli P Salazar de Pablo G Correll CU Meyer-Lindenberg A Millan MJ Borgwardt S . Prevention of psychosis: advances in detection, prognosis, and intervention. JAMA Psychiatry. (2020) 77:755–65. doi: 10.1001/jamapsychiatry.2019.4779. PMID: 32159746

[19] Davies C Cipriani A Ioannidis JPA Radua J Stahl D Provenzani U . Lack of evidence to favor specific preventive interventions in psychosis: a network meta-analysis. World Psychiatry. (2018) 17:196–209. doi: 10.1002/wps.20526. PMID: 29856551 PMC5980552

[20] van Os J Guloksuz S . A critique of the “ultra-high risk” and “transition” paradigm. World Psychiatry. (2017) 16:200–6. doi: 10.1002/wps.20423. PMID: 28498576 PMC5428198

[21] National Institute for Health and Care Excellence . Psychosis and schizophrenia in adults: prevention and management. NICE Clinical Guideline CG178. London: NICE (2014). 31869042

[22] Abidi S Mian I Garcia-Ortega I Lecomte T Raedler T Jackson K . Canadian guidelines for the pharmacological treatment of schizophrenia spectrum and other psychotic disorders in children and youth. Can J Psychiatry. (2017) 62:635–47. doi: 10.1177/0706743717720197. PMID: 28764561 PMC5593251

[23] National Institute for Health and Care Excellence . Psychosis and schizophrenia in adults: quality standard [QS80. London: NICE (2015). Available online at: https://www.nice.org.uk/guidance/qs80 (Accessed May 20, 2026).

[24] Institute of Medicine . Clinical practice guidelines we can trust. Washington, DC: National Academies Press (2011).

[25] Campbell SM Braspenning J Hutchinson A Marshall M . Research methods used in developing and applying quality indicators in primary care. Qual Saf Health Care. (2002) 11:358–64. doi: 10.1136/qhc.11.4.358. PMID: 12468698 PMC1758017

[26] Galletly C Castle D Dark F Humberstone V Jablensky A Killackey E . Royal Australian and New Zealand College of Psychiatrists clinical practice guidelines for the management of schizophrenia and related disorders. Aust N Z J Psychiatry. (2016) 50:410–72. doi: 10.1177/0004867416641195. PMID: 27106681

[27] Alonso-Coello P Irfan A Sola I Gich I Delgado-Noguera M Rigau D . The quality of clinical practice guidelines over the last two decades: a systematic review of guideline appraisal studies. Qual Saf Health Care. (2010) 19:e58. doi: 10.1136/qshc.2010.042077. PMID: 21127089

[28] Kung J Miller RR Mackowiak PA . Failure of clinical practice guidelines to meet Institute of Medicine standards: two more decades of little, if any, progress. Arch Intern Med. (2012) 172:1628–33. doi: 10.1001/2013.jamainternmed.56. PMID: 23089902

[29] Chen Y Yang K Marušic A Qaseem A Meerpohl JJ Flottorp S . A reporting tool for practice guidelines in health care: the RIGHT statement. Ann Intern Med. (2017) 166:128–32. doi: 10.7326/M16-1565. PMID: 27893062

[30] Brouwers MC Kho ME Browman GP Burgers JS Cluzeau F Feder G . AGREE II: advancing guideline development, reporting and evaluation in health care. CMAJ. (2010) 182:E839–42. doi: 10.1503/cmaj.090449. PMID: 20603348 PMC3001530

[31] Gaebel W Großimlinghaus I Heuser P Janssen B Johnson B Kurimay T . European Psychiatric Association (EPA) guidance on quality assurance in mental health care. Eur Psychiatry. (2015) 30:360–87. doi: 10.1016/j.eurpsy.2014.11.003. PMID: 25725593

[32] Barbui C Girlanda F Ay E Cipriani A Becker T Koesters M . Implementation of treatment guidelines for specialist mental health care. Cochrane Database Syst Rev. (2014) 17(1):CD009780. doi: 10.1002/14651858.CD009780.pub2. PMID: 24443146

[33] Zhu Y Xu L Wang H Liu Y Wu J Zhou C . The quality of clinical practice guidelines in China: a systematic assessment using the AGREE II instrument. PloS One. (2012) 7:e40864. doi: 10.1371/journal.pone.0040864. PMID: 22870205 PMC3411599

[34] Page MJ McKenzie JE Bossuyt PM Boutron I Hoffmann TC Mulrow CD . The PRISMA 2020 statement: an updated guideline for reporting systematic reviews. BMJ. (2021) 372:n71. doi: 10.1136/bmj.n71. PMID: 33782057 PMC8005924

[35] Scognamiglio P . Early intervention in psychosis guidelines – AGREE II systematic review. Charlottesville, VA: Center for Open Science / OSF Registries (2025). Available online at: https://osf.io/cek7u (Accessed May 20, 2026).

[36] Brosseau L Rahman P Toupin-April K Poitras S King J De Angelis G . A systematic critical appraisal of non-pharmacological management of osteoarthritis using the AGREE II instrument and expert consensus. PloS One. (2014) 9:e95369. doi: 10.1371/journal.pone.0095369. PMID: 24840205 PMC4026323

[37] Addington J Addington D Abidi S Raedler T Remington G . Canadian treatment guidelines for individuals at clinical high risk of psychosis. Can J Psychiatry. (2017) 62:656–61. doi: 10.1177/0706743717719895. PMID: 28730848 PMC5593244

[38] American Psychiatric Association . The American Psychiatric Association practice guideline for the treatment of patients with schizophrenia. Washington, DC: American Psychiatric Association Publishing (2020). doi: 10.1176/appi.books.9780890424841 PMC7725162 33343262

[39] Department of Veterans Affairs, Department of Defense . VA/DoD clinical practice guideline for management of first-episode psychosis and schizophrenia. Washington, DC: U.S. Government Printing Office (2023).

[40] Asian Network of Early Psychosis Writing Group . Early psychosis declaration for Asia by the Asian Network of Early Psychosis. East Asian Arch Psychiatry. (2012) 22:90–3. 23019280

[41] British Columbia Ministry of Health Services . Standards and guidelines for early psychosis intervention (EPI) programs. Victoria (BC: Ministry of Health Services (2010). p. 106. Available online at: https://www.bcchildrens.ca/sites/g/files/qpdaav156/files/2024-12/BC-EPI-standards-guidelines.pdf (Accessed May 20, 2026).

[42] Bromley S Choi MA Faruqui S . First episode psychosis: an information guide. In: Revised ed. Centre for Addiction and Mental Health, Toronto (ON (2015).

[43] De Masi S Sampaolo L Mele A Morciano C Cappello S Meneghelli A . The Italian guidelines for early intervention in schizophrenia: development and conclusions. Early Interv Psychiatry. (2008) 2:291–302. doi: 10.1111/j.1751-7893.2008.00091.x. PMID: 21352163

[44] Health Service Executive (HSE) . Early intervention in psychosis: model of care. Dublin: HSE (2019). Available online at: https://www.hse.ie/eng/about/who/cspd/ncps/mental-health/psychosis/resources/hse-early-intervention-in-psychosis-model-of-care-june-20191.pdf (Accessed May 20, 2026).

[45] International Early Psychosis Association Writing Group . International clinical practice guidelines for early psychosis. Br J Psychiatry Suppl. (2005) 187(Suppl 48):s120–4. doi: 10.1192/bjp.187.48.s120. PMID: 16055801

[46] IRIS . IRIS Guidelines Update September 2012: Revision of the Original 1998 IRIS Guidelines. London: IRIS Initiative Ltd (2012). Available online at: https://iris-initiative.org.uk/silo/files/iris-guidelines-update--september-2012.pdf (Accessed May 20, 2026).

[47] Lecomte T Abidi S Garcia-Ortega I Mian I Jackson K Pryor D . Canadian treatment guidelines on psychosocial treatment of schizophrenia in children and youth. Can J Psychiatry. (2017) 62:648–55. doi: 10.1177/0706743717720195. PMID: 28886670 PMC5593249

[48] Lo TL Warden M He Y Si T Kalyanasundaram S Thirunavukarasu M . Recommendations for the optimal care of patients with recent‐onset psychosis in the Asia‐Pacific region. Asia-Pacific Psychiatry. (2016) 8:154–71. doi: 10.1111/appy.12234. PMID: 27062665 PMC4834614

[49] Mead K Greer T . Best Practices and Challenges in First Episode Psychosis Care: Implications for the UTSW Psychosis Center. Dallas, TX: Meadows Mental Health Policy Institute (2016). Available online at: https://mmhpi.org/wp-content/uploads/2016/12/FEPCare_BestPractice_Implications_for_UTSWPsychosisCntr_Final.pdf (Accessed May 20, 2026).

[50] National Institute for Health and Care Excellence (NICE) . Psychosis and schizophrenia in children and young people: recognition and management. Clinical guideline CG155. London: NICE (2013). Available online at: https://www.nice.org.uk/guidance/cg155 (Accessed May 20, 2026).

[51] NHS England . Implementing the Early Intervention in Psychosis access and waiting time standard. London: NHS England (2023). Available online at: https://www.england.nhs.uk/wp-content/uploads/2023/03/B1954-implementing-the-early-intervention-in-psychosis-access-and-waiting-time-standard.pdf (Accessed May 20, 2026).

[52] Ontario Ministry of Health and Long-Term Care . Early psychosis intervention program standards. Toronto (ON: Ministry of Health and Long-Term Care (2011). Available online at: https://cmhaww.ca/wp-content/uploads/2023/10/epi_program_standards-2011.pdf (Accessed May 20, 2026).

[53] Oregon Early Assessment and Support Alliance (EASA) . Practice guidelines for Oregon EASA. Salem, OR: Oregon Health Authority (2014). Available online at: https://www.oregon.gov/oha/HSD/AMH/Reporting%20Reqs/Practice%20guidelines%20for%20Oregon%20EASA.pdf (Accessed May 20, 2026).

[54] Orygen Youth Health . Australian clinical guidelines for early psychosis. Melbourne: Orygen Youth Health (2010). Available online at: https://www.orygen.org.au/Campus/Expert-Network/Resources/Free/Clinical-Practice/Australian-Clinical-Guidelines-for-Early-Psychosis/Australian-Clinical-Guidelines-for-Early-Psychosis.aspx (Accessed May 20, 2026).

[55] Royal College of Psychiatrists . Standards for early intervention in psychosis services. London: RCPsych (2021). Available online at: https://www.rcpsych.ac.uk/docs/default-source/improving-care/ccqi/quality-networks/early-intervention-in-psychosis-teams-(eipn)/epin-standards-first-edition.pdf (Accessed May 20, 2026).

[56] Schmidt SJ Schultze-Lutter F Schimmelmann BG Maric NP Salokangas RKR Riecher-Rössler A . EPA guidance on the early intervention in clinical high risk states of psychoses. Eur Psychiatry. (2015) 30:388–404. doi: 10.1016/j.eurpsy.2015.01.013. PMID: 25749390

[57] Schultze-Lutter F Michel C Schmidt SJ Schimmelmann BG Maric NP Salokangas RKR . EPA guidance on the early detection of clinical high risk states of psychoses. Eur Psychiatry. (2015) 30:405–16. doi: 10.1016/j.eurpsy.2015.01.010. PMID: 25735810

[58] Scottish Intercollegiate Guidelines Network (SIGN) . Management of schizophrenia: a national clinical guideline. Edinburgh: SIGN (2013). Available online at: https://www.sign.ac.uk/assets/sign131.pdf (Accessed May 20, 2026).

[59] Working Group of the Clinical Practice Guideline for Schizophrenia and Incipient Psychotic Disorder; Mental Health Forum . Clinical Practice Guideline for Schizophrenia and Incipient Psychotic Disorder. Madrid: Quality Plan for the National Health System of the Ministry of Health and Consumer Affairs; Agency for Health Technology Assessment and Research (2009). Clinical Practice Guideline: CAHTA Number 2006/05-2.

[60] Williams R Ostinelli EG Agorinya J Minichino A De Crescenzo F Maughan D . Comparing interventions for early psychosis: a systematic review and component network meta-analysis. EClinicalMedicine. (2024) 70:102537. doi: 10.1016/j.eclinm.2024.102537. PMID: 38516103 PMC10955207

[61] Williams R Penington E Gupta V Quirk A Tsiachristas A Rickett M . Critical components of ‘Early Intervention in Psychosis’: national retrospective cohort study. Br J Psychiatry. (2025) 26:1–9. doi: 10.1192/bjp.2025.126. PMID: 40566951 PMC7617821

[62] Poletti M Pelizza L Preti A Raballo A . Clinical High-Risk for Psychosis (CHR-P) circa 2024: synoptic analysis and synthesis of contemporary treatment guidelines. Asian J Psychiatr. (2024) 100:104142. doi: 10.1016/j.ajp.2024.104142. PMID: 39083954

[63] Minichino A Davies C Karpenko O Oliver D O’Donoghue B McGorry P . Preventing psychosis in people at clinical high risk: an updated meta-analysis by the World Psychiatric Association Preventive Psychiatry section. Mol Psychiatry. (2025) 30:2773–82. doi: 10.1038/s41380-025-02902-8. PMID: 39953286 PMC12092282

[64] O’Connell N O’Connor K McGrath D Vagge L Mockler D Jennings R . Early Intervention in Psychosis services: a systematic review and narrative synthesis of the barriers and facilitators to implementation. Eur Psychiatry. (2021) 65:e2. doi: 10.1192/j.eurpsy.2021.2260. PMID: 34913421 PMC8792869

[65] Dehbozorgi R Fereidooni-Moghadam M Shahriari M Moghimi-Sarani E . A quality assessment of clinical practice guidelines with recommendations for family involvement in the care of individuals diagnosed with schizophrenia, bipolar mood disorder, and major depressive disorder: critical appraisal utilizing AGREE II. Front Psychiatry. (2023) 13:1065129. doi: 10.3389/fpsyt.2022.1065129. PMID: 36683976 PMC9845625

[66] Thoonsen AC van Schoten SM Merten H van Beusekom I Schoonmade LJ Delnoij DMJ . Stimulating implementation of clinical practice guidelines in hospital care from a central guideline organization perspective: a systematic review. Health Policy. (2024) 148:105135. doi: 10.1016/j.healthpol.2024.105135. PMID: 39128438

[67] Scognamiglio P Morena D Di Fazio N Delogu G Iniziato V La Pia S . Vox clamantis in deserto: a survey among Italian psychiatrists on defensive medicine and professional liability. Front Psychiatry. (2023) 14:1244101. doi: 10.3389/fpsyt.2023.1244101. PMID: 37663598 PMC10469623

[68] Studdert DM Mello MM Sage WM DesRoches CM Peugh J Zapert K . Defensive medicine among high-risk specialist physicians in a volatile malpractice environment. JAMA. (2005) 293:2609–17. doi: 10.1001/jama.293.21.2609. PMID: 15928282

[69] Recupero PR Rainey SE . Forensic psychiatry and defensive medicine. J Am Acad Psychiatry Law. (2010) 38:544–54.

[70] Regione Emilia-Romagna. Direzione Generale Cura della persona, salute e welfare . Linee di indirizzo per la promozione della salute e del benessere nelle persone alla prima manifestazione psicotica o ad alto rischio di psicosi. Bologna: Regione Emilia-Romagna (2024). Available online at: https://salute.regione.emilia-romagna.it/salute-mentale/documentazione/linee_di_indirizzo_esordi_psicotici_2024_rev3.pdf/@@download/file/Linee_di_indirizzo_Esordi_psicotici_2024_rev3.pdf (Accessed May 20, 2026).

