# Supplementary material for: Clinical practice guidelines and quality standards for early intervention in psychosis: an AGREE II appraisal and systematic review of service components
Source: Front Psychiatry. 2026 Jun 3;17:1831668. doi: 10.3389/fpsyt.2026.1831668 (PMC13272451; doi:10.3389/fpsyt.2026.1831668)
Supplement: Supplementary file 1 [file Table1.docx]

**Supplementary Table S1. PRISMA 2020 checklist for the present systematic review.**

| **Section and topic** | **Item #** | **Checklist item** | **Location where item is reported** |
| --- | --- | --- | --- |
| **TITLE** | | | |
| Title | 1 | Identify the report as a systematic review. | Title. |
| **ABSTRACT** | | | |
| Abstract | 2 | See the PRISMA 2020 for Abstracts checklist. | Abstract. |
| **INTRODUCTION** | | | |
| Introduction | 3 | Describe the rationale for the review in the context of existing knowledge. | Introduction, paragraphs describing the youth mental health context, early intervention in psychosis, guideline heterogeneity, and AGREE II rationale. |
| Introduction | 4 | Provide an explicit statement of the objective(s) or question(s) the review addresses. | Introduction, final paragraph, where the review questions and objectives are stated. |
| **METHODS** | | | |
| Methods | 5 | Specify the inclusion and exclusion criteria for the review and how studies were grouped for the syntheses. | Methods, section 2.2 Eligibility criteria; population-specific grouping for CHR-P and FEP is described in section 2.4 Data extraction and synthesis of recommendations. |
| Methods | 6 | Specify all databases, registers, websites, organisations, reference lists and other sources searched or consulted. Specify the date when each source was last searched or consulted. | Methods, section 2.1 Study design, registration, and search strategy; complete source list, dates of consultation, browsing approaches, and retrieval details are reported in Supplementary Table S5. |
| Methods | 7 | Present the full search strategies for all databases, registers and websites, including any filters and limits used. | Methods, section 2.1 Study design, registration, and search strategy; full source-specific search strategies for databases and grey-literature sources are reported in Supplementary Table S5. |
| Methods | 8 | Specify the methods used to decide whether a study met the inclusion criteria of the review, including how many reviewers screened each record and each report retrieved, whether they worked independently, and, if applicable, details of automation tools used in the process. | Methods, section 2.1 Study design, registration, and search strategy, and section 2.4 Data extraction and synthesis of recommendations: two-stage screening, reviewer workflow, duplicate procedures, and absence of automation tools are reported. |
| Methods | 9 | Specify the methods used to collect data from reports, including how many reviewers collected data from each report, whether they worked independently, any processes for obtaining or confirming data from study investigators, and, if applicable, details of automation tools used in the process. | Methods, section 2.4 Data extraction and synthesis of recommendations. |
| Methods | 10 | Specify any methods used to measure risk of bias in the included studies. | Not applicable. This review appraised methodological quality of CPGs and QSs using AGREE II, as described in Methods, section 2.3 Guideline appraisal; no primary-study risk-of-bias assessment was conducted. |
| Methods | 11 | Specify the methods used to present and synthesize results of the studies. | Methods, section 2.4 Data extraction and synthesis of recommendations. |
| Methods | 12 | Specify any methods used to explore possible causes of heterogeneity among study results (e.g., subgroup analysis or meta-regression). | Not applicable. No quantitative synthesis or meta-analysis was performed; document heterogeneity was handled descriptively in Methods, section 2.4, and Results, section 3.5. |
| Methods | 13 | Specify any sensitivity analyses conducted to assess robustness of the synthesized results. | Sensitivity analyses are described in Methods, section 2.4 Data extraction and synthesis of recommendations, and reported in Results, section 3.5 Patterns in the frequency and strength of recommendations. |
| Methods | 14 | Describe methods of synthesizing any additional analyses (e.g., scoping reviews, economic models). | Not applicable. No additional analyses of the type specified were conducted. |
| **RESULTS** | | | |
| Results | 15 | Describe the results of the search and selection process, including reasons for exclusion of studies considered but not meeting inclusion criteria, ideally using a flow diagram. | Results, section 3.1 Study selection; Figure 1 PRISMA 2020 flow diagram. |
| Results | 16a | Cite each included study and present its characteristics. | Included documents are described in Results, section 3.2 Characteristics of included guidelines and quality standards, and in main manuscript Table 1. |
| Results | 16b | Cite each excluded study and present the primary reason for exclusion. | The full list of excluded full-text reports with primary reasons for exclusion is provided in Supplementary Table S6, in accordance with PRISMA 2020 item 16b. |
| Results | 17 | Cite each included study and present its results. | Results, sections 3.3 Methodological quality of guidelines, 3.4 Core components of youth early intervention in psychosis services, and 3.5 Patterns in the frequency and strength of recommendations; main manuscript Tables 2 and 3; Supplementary Tables S3 and S4. |
| Results | 18 | Present results of all statistical syntheses. If meta-analysis was done, present for each the summary estimate and its precision, along with measures of statistical heterogeneity. | Not applicable. No meta-analysis was conducted. Descriptive AGREE II results are reported in Results, section 3.3 Methodological quality of guidelines, and in main manuscript Table 2. |
| Results | 19 | Present results of any investigations of possible causes of heterogeneity among study results. | Not applicable. No formal investigation of causes of heterogeneity among quantitative study results was conducted. |
| Results | 20 | Present results of any sensitivity analyses. | Results, section 3.5 Patterns in the frequency and strength of recommendations. |
| Results | 21 | Provide a brief summary of the characteristics and risk of bias among the included studies. | Characteristics are reported in Results, section 3.2, and main manuscript Table 1; methodological quality is reported in Results, section 3.3, and main manuscript Table 2; methodological limitations are discussed in Discussion, section 4.5 Strengths and limitations. |
| Results | 22 | Provide a brief summary of results of each synthesis, the overall strength of evidence for each outcome, and, for meta-analyses, a description of the interpretation of the results in a clinical or policy context. | Results, sections 3.4 Core components of youth early intervention in psychosis services and 3.5 Patterns in the frequency and strength of recommendations; main manuscript Table 3; Discussion, sections 4.1-4.4. |
| Results | 23 | Provide a brief summary of the results of any assessments of certainty (or confidence) in the body of evidence for each outcome. | Formal certainty assessment of primary-study evidence was not performed. Limitations of the harmonized recommendation-strength framework are discussed in Discussion, section 4.5 Strengths and limitations. |
| **DISCUSSION** | | | |
| Discussion | 24a | Provide a general interpretation of the results in the context of other evidence. | Discussion, sections 4.1 Summary of main findings, 4.2 Methodological quality of early psychosis guidelines, and 4.3 Core components of EIP services. |
| Discussion | 24b | Discuss any limitations of the evidence included in the review. | Discussion, section 4.5 Strengths and limitations. |
| Discussion | 24c | Discuss any limitations of the review processes used. | Discussion, section 4.5 Strengths and limitations. |
| Discussion | 24d | Discuss implications of the results for practice, policy, and future research. | Discussion, section 4.4 Implications for policy, practice, and research; Conclusion. |
| **OTHER INFORMATION** | | | |
| Other Information | 24a | Provide registration information for the review, including register name and registration number, or state that the review was not registered. | Methods, section 2.1 Study design, registration, and search strategy: OSF registration reported (cek7u). |
| Other Information | 24b | Indicate where the review protocol can be accessed, or state that a protocol was not prepared. | Methods, section 2.1 Study design, registration, and search strategy, and reference list entry for the OSF registry record. |
| Other Information | 24c | Describe and explain any amendments to information provided at registration or in the protocol. | Methods, section 2.1 Study design, registration, and search strategy, and section 2.4 Data extraction and synthesis of recommendations: post hoc sensitivity analyses are explicitly identified as post hoc. |
| Other Information | 25 | Describe sources of financial or non-financial support for the review, and the role of the funders or sponsors in the review. | Funding section: no financial support reported. |
| Other Information | 26 | Declare any competing interests of review authors. | Conflict of Interest section. |
| Other Information | 27 | Report which of the following are publicly available and where they can be found: template data collection forms; data extracted from included studies; data used for all analyses; analytic code; any other materials used in the review. | Supplementary Tables S2-S4 and S6; additional data extraction tables and analytic decision rules are available from the corresponding author upon reasonable request. |

The table reports the PRISMA 2020 items, the corresponding reporting status, and the manuscript section or supplementary-table location where each item is addressed. PRISMA items were interpreted in relation to a systematic review of clinical practice guidelines and quality standards rather than primary intervention studies; where relevant, item reporting was adapted to this document-based review design.
