# Supplementary material for: Clinical practice guidelines and quality standards for early intervention in psychosis: an AGREE II appraisal and systematic review of service components
Source: Front Psychiatry. 2026 Jun 3;17:1831668. doi: 10.3389/fpsyt.2026.1831668 (PMC13272451; doi:10.3389/fpsyt.2026.1831668)
Supplement: Supplementary file 2 [file Table2.docx]

**Supplementary Table S1A. Intraclass correlation coefficients (ICC) for AGREE II domain scores.**

| **AGREE II domain** | **Appraiser 1 mean (SD)** | **Appraiser 2 mean (SD)** | **ICC (2,1)** | **95% CI** | **Interpretation** |
| --- | --- | --- | --- | --- | --- |
| Scope and purpose | 90.2 (9.8) | 91.0 (10.7) | 0.87 | 0.82–0.91 | Good |
| Stakeholder involvement | 74.9 (14.3) | 76.1 (13.8) | 0.83 | 0.77–0.88 | Good |
| Rigour of development | 62.1 (22.6) | 63.4 (21.9) | 0.89 | 0.85–0.93 | Excellent |
| Clarity of presentation | 83.6 (12.1) | 84.0 (11.6) | 0.85 | 0.80–0.89 | Good |
| Applicability | 63.3 (19.7) | 64.1 (20.2) | 0.82 | 0.76–0.87 | Good |
| Editorial independence | 61.5 (34.6) | 62.1 (35.0) | 0.91 | 0.87–0.94 | Excellent |
| Overall AGREE II index | 72.6 (13.4) | 73.5 (13.1) | 0.88 | 0.84–0.92 | Good |

*Two-way mixed-effects model, absolute agreement, single measures. Interpretation: <0.50 = poor; 0.50–0.75 = moderate; 0.75–0.90 = good; >0.90 = excellent (Koo TK, Mae AY. J Chiropr Med. 2016;15(2):155–163). SD = standard deviation; ICC = intraclass correlation coefficient.*

**Supplementary Table S1B. Cohen's kappa (κ) for inter-rater agreement at the title/abstract and full-text screening stages.**

| **Screening stage** | **Total items screened** | **Items in disagreement** | **Cohen's κ** | **95% CI** | **Interpretation** |
| --- | --- | --- | --- | --- | --- |
| Title/abstract screening (n = 2,592) | 2,592 | 87 (3.4%) | 0.83 | 0.79–0.87 | Strong |
| Full-text eligibility assessment (n = 582) | 582 | 38 (6.5%) | 0.88 | 0.83–0.93 | Strong |

*κ values ≥0.80 are interpreted as strong agreement (Landis & Koch, 1977). Disagreements were resolved through discussion between the two reviewers; residual disagreements were adjudicated by the senior author.*
