# Supplementary material for: Clinical practice guidelines and quality standards for early intervention in psychosis: an AGREE II appraisal and systematic review of service components
Source: Front Psychiatry. 2026 Jun 3;17:1831668. doi: 10.3389/fpsyt.2026.1831668 (PMC13272451; doi:10.3389/fpsyt.2026.1831668)
Supplement: Supplementary file 3 [file Table3.docx]

**Supplementary Table S2. Crosswalk of original recommendation grading systems and harmonized three-level framework.**

| **Document abbreviation** | **Document title** | **Explicit source grading** | **Original grading system / wording** | **Mapped to strong/mandatory** | **Mapped to moderate/recommended** | **Mapped to weak/optional** | **Notes** |
| --- | --- | --- | --- | --- | --- | --- | --- |
| ISS-SNLG | The Italian Guidelines for Early Intervention in Schizophrenia | Yes | Italian recommendation classes (IA, IB, IIB, IIIB, VIB, IC) | IA | IB; IIB; IIIB; VIB | IC | Document treated as explicitly graded in the synthesis. |
| MHS Catalunya | Clinical Practice Guideline for Schizophrenia and Incipient Psychotic Disorder | Yes | Recommendation grades A–C | A | B | C | Document treated as explicitly graded in the synthesis. |
| SIGN | Management of Schizophrenia | Yes | SIGN grades A–D plus Good Practice Points (GPP) | A | B; C | D; GPP | Same operational rule also applied to EPA and CPA adapted recommendations when those source grades were retained. |
| EPA Intervention | EPA Guidance on the Early Intervention of Clinical High Risk States of Psychosis | Yes | EPA guidance using SIGN-style grades A–D plus GPP | A | B; C | D; GPP | Grouped with SIGN-style systems in the prior extraction. |
| EPA Detection | EPA Guidance on the Early Detection of Clinical High Risk States of Psychosis | Yes | EPA guidance using SIGN-style grades A–D plus GPP | A | B; C | D; GPP | Grouped with SIGN-style systems in the prior extraction. |
| CPA CHR-P | Canadian Treatment Guidelines for Individuals at Clinical High Risk of Psychosis | Yes | Adapted guideline preserving source grades; operationally treated as SIGN/EPA-style A–D plus GPP where applicable | A | B; C | D; GPP | Recommendations were adapted from NICE/EPA source guidance, but grades were retained and harmonized at extraction level. |
| CPA FEP | Canadian Treatment Guidelines on Psychosocial Treatment of Schizophrenia in Children and Youth | Yes | Adapted guideline preserving source grades; operationally treated as SIGN/EPA-style A–D plus GPP where applicable | A | B; C | D; GPP | Recommendations were adapted from existing source guidelines and harmonized at extraction level. |
| NICE CG 178 | Psychosis and Schizophrenia in Adults: Treatment and Management | Yes | Directive wording: must/must not; offer/do not offer; consider | must; must not; offer; do not offer | consider | – | Negative directive wording (e.g., do not offer) was handled as strong/mandatory negative recommendation. |
| NICE CG 155 | Psychosis and Schizophrenia in Children and Young People: Recognition and Management | Yes | Directive wording: must/must not; offer/do not offer; consider | must; must not; offer; do not offer | consider | – | Same operational rule as NICE CG 178. |
| RANZCP | Clinical Practice Guidelines for the Management of Schizophrenia and Related Disorders | Yes | EBR I; EBR II; EBR III-1; EBR III-2; EBR IV; CBR III-1; CBR | EBR I; EBR II; EBR III-1 | EBR III-2 | EBR IV; CBR III-1; CBR | Consensus-based recommendation codes (CBR) were operationalized as weak/optional for harmonization. |
| Orygen | Australian Clinical Guidelines for Early Psychosis – 2nd ed. | Yes | Recommendation grades A–D plus Good Practice Points (GPP) | A; B | C | D; GPP | Operational rule carried forward from the prior extraction table. |
| RCPsych | Standards for Early Intervention in Psychosis Services | Yes | Type 1; Type 2; Type 3 standards | Type 1 | Type 2 | Type 3 | Type 1 = essential/fundamental; Type 2 = expected; Type 3 = desirable/aspirational. |
| APA | The American Psychiatric Association Practice Guideline for the Treatment of Patients with Schizophrenia | Yes | Strength of recommendation + evidence rating; operationalized from extracted combinations 1A/1B/1C and 2B/2C | 1A; 1B; 1C | 2B; 2C | – | The APA system does not have a separate weak/optional tier; extracted 'recommendation' statements were treated as strong and 'suggestion' statements as moderate. |
| VA–DoD | VA/DoD Clinical Practice Guideline for Management of First-Episode Psychosis and Schizophrenia | Yes | Strength and direction: Strong for; Weak for; Neither for nor against; Weak against; Strong against | Strong for; Strong against | Weak for; Weak against | – | 'Neither for nor against' was not counted as an endorsement and was therefore not mapped to a positive/negative strength cell in Table 3. |
| ANEP | Early Psychosis Declaration for Asia | No | Narrative / consensus statements without explicit recommendation-strength taxonomy | – | – | – | Classified as not graded in the synthesis. |
| MHS British Columbia | Standards and Guidelines for Early Psychosis Intervention (EPI) Programs | No | Narrative / consensus statements without explicit recommendation-strength taxonomy | – | – | – | Classified as not graded in the synthesis. |
| CAMH | First Episode Psychosis: An Information Guide | No | Narrative / informational guidance without explicit recommendation-strength taxonomy | – | – | – | Classified as not graded in the synthesis. |
| HSE | National Clinical Programme for Early Intervention in Psychosis – Model of Care | No | Narrative / model-of-care statements without explicit recommendation-strength taxonomy | – | – | – | Classified as not graded in the synthesis. |
| IEPA | International Clinical Practice Guidelines for Early Psychosis | No | Narrative / consensus recommendations without explicit recommendation-strength taxonomy | – | – | – | Classified as not graded in the synthesis. |
| IRIS | Early Intervention in Psychosis Guidelines Update | No | Narrative / consensus recommendations without explicit recommendation-strength taxonomy | – | – | – | Classified as not graded in the synthesis. |
| NICE QS 80 | Psychosis and Schizophrenia in Adults – Quality Standard No. 80 | No | Quality statements without a separate recommendation-strength taxonomy | – | – | – | Retained in the review but classified as not graded for Table 3. |
| APP | Recommendations for the Optimal Care of Patients with Recent-Onset Psychosis in the Asia-Pacific Region | No | Narrative / consensus recommendations without explicit recommendation-strength taxonomy | – | – | – | Classified as not graded in the synthesis. |
| MMHPI | Best Practices in First Episode Psychosis Care: Implications for the UTSW Psychosis Center | No | Narrative / best-practice statements without explicit recommendation-strength taxonomy | – | – | – | Classified as not graded in the synthesis. |
| NCCMH–NICE | Implementing the Early Intervention in Psychosis Access and Waiting Time Standard | No | Implementation guidance without a separate recommendation-strength taxonomy | – | – | – | Classified as not graded in the synthesis. |
| MH Ontario | Early Psychosis Intervention Program Standards | No | Standards statements without explicit recommendation-strength taxonomy | – | – | – | Classified as not graded in the synthesis. |
| EASA | Practice Guidelines for Oregon EASA Programs | No | Practice guidance without explicit recommendation-strength taxonomy | – | – | – | Classified as not graded in the synthesis. |

*This table shows how the original recommendation grading systems used across included clinical practice guidelines and quality standards were mapped onto the harmonized three-level framework applied in the review (strong/mandatory, moderate/recommended, weak/optional). For documents without an explicit recommendation-strength taxonomy, this is indicated separately as not graded.*
