# Supplementary material for: Clinical practice guidelines and quality standards for early intervention in psychosis: an AGREE II appraisal and systematic review of service components
Source: Front Psychiatry. 2026 Jun 3;17:1831668. doi: 10.3389/fpsyt.2026.1831668 (PMC13272451; doi:10.3389/fpsyt.2026.1831668)
Supplement: Supplementary file 4 [file Table4.docx]

**Supplementary Table S3. Full document-level recommendation matrix for clinical high risk for psychosis (CHR-P).**

| **Population** | **Domain** | **Recurrent service component** | **Documents endorsing, n/N (%)** | **Strong/mandatory documents** | **Moderate/recommended documents** | **Weak/optional documents** | **Endorsed but not graded documents** |
| --- | --- | --- | --- | --- | --- | --- | --- |
| **CHR-P** | Service configuration and organization | Specialized multidisciplinary CHR-P services / dedicated pathways within youth mental health or EIP teams | 16/17 (94.1) | NICE CG 155; RANZCP; CPA CHR-P; RCPsych | NICE CG 178; Orygen | ISS-SNLG; EPA Detection; VA-DoD | MHS British Columbia; HSE; IEPA; IRIS; NCCMH-NICE; MH Ontario; EASA |
| **CHR-P** | Service configuration and organization | Low-threshold and inclusive access routes | 7/17 (41.2) | NICE CG 155 | NICE CG 178; RCPsych | Orygen | HSE; MH Ontario; EASA |
| **CHR-P** | Service configuration and organization | Integration with primary care and other mental health services | 11/17 (64.7) | NICE CG 178; NICE CG 155; CPA CHR-P | ISS-SNLG; RCPsych | Orygen; VA-DoD | HSE; IEPA; NCCMH-NICE; MH Ontario |
| **CHR-P** | Service configuration and organization | Proactive outreach and early detection activities | 8/17 (47.1) | RANZCP | ISS-SNLG; Orygen |  | HSE; IEPA; IRIS; MH Ontario; EASA |
| **CHR-P** | Service configuration and organization | Youth-friendly, low-stigma environments† | 12/17 (70.6) |  | ISS-SNLG; RCPsych | EPA Intervention; RANZCP | MHS British Columbia; HSE; IEPA; IRIS; NCCMH-NICE; MH Ontario; EASA; CPA CHR-P |
| **CHR-P** | Assessment | Comprehensive multidisciplinary biopsychosocial assessment | 14/17 (82.4) | NICE CG 155; RANZCP; CPA CHR-P; RCPsych | ISS-SNLG; NICE CG 178; EPA Detection; EPA Intervention | Orygen | MHS British Columbia; HSE; IEPA; IRIS; MH Ontario |
| **CHR-P** | Assessment | Routine assessment of psychiatric and substance-use comorbidities | 12/17 (70.6) | NICE CG 178; NICE CG 155; RANZCP; CPA CHR-P | RCPsych | EPA Intervention; Orygen | HSE; IEPA; IRIS; MH Ontario; NCCMH-NICE |
| **CHR-P** | Assessment | Structured assessment of functioning and role performance | 11/17 (64.7) | NICE CG 178; RANZCP; RCPsych | EPA Detection; EPA Intervention | Orygen; CPA CHR-P; VA-DoD | HSE; IEPA; MH Ontario |
| **CHR-P** | Assessment | Validated instruments / structured interviews for CHR-P identification and diagnostic formulation | 10/17 (58.8) | NICE CG 178; CPA CHR-P; RCPsych | EPA Detection; EPA Intervention | ISS-SNLG; RANZCP; VA-DoD | IEPA; MHS British Columbia |
| **CHR-P** | Treatment | CBT-based psychological interventions | 12/17 (70.6) | NICE CG 178; EPA Intervention; RANZCP; CPA CHR-P; RCPsych | NICE CG 155; ISS-SNLG | Orygen | HSE; IEPA; IRIS; MHS British Columbia |
| **CHR-P** | Treatment | Family-focused interventions | 12/17 (70.6) | NICE CG 178; RANZCP; CPA CHR-P; RCPsych; VA-DoD | NICE CG 155; Orygen | ISS-SNLG | HSE; IEPA; IRIS; MHS British Columbia |
| **CHR-P** | Treatment | Psychoeducation | 6/17 (35.3) | RANZCP | Orygen |  | HSE; IEPA; IRIS; NCCMH-NICE |
| **CHR-P** | Treatment | Avoidance of routine antipsychotic use as first-line strategy in CHR-P | 13/17 (76.5) | NICE CG 155; RANZCP | NICE CG 178; Orygen | ISS-SNLG; EPA Intervention; CPA CHR-P | MHS British Columbia; HSE; IEPA; IRIS; MH Ontario; EASA |

*This table presents the complete document-level matrix for recurrent CHR-P service components, showing which included guidance documents endorsed each component and how each endorsement was classified within the harmonized recommendation-strength framework (strong/mandatory, moderate/recommended, weak/optional, or endorsed but not graded). It provides the document-level basis underlying the aggregate synthesis reported in Table 3.*

*† Youth-friendly, low-stigma environments: met the frequency criterion (12/17, 70.6%) but had no strong or mandatory recommendation and therefore did not qualify as a core component under the strict prespecified rule; retained here as a descriptively endorsed component.*
