# Supplementary material for: Clinical practice guidelines and quality standards for early intervention in psychosis: an AGREE II appraisal and systematic review of service components
Source: Front Psychiatry. 2026 Jun 3;17:1831668. doi: 10.3389/fpsyt.2026.1831668 (PMC13272451; doi:10.3389/fpsyt.2026.1831668)
Supplement: Supplementary file 5 [file Table5.docx]

**Supplementary Table S4. Full document-level recommendation matrix for first-episode psychosis (FEP).**

| **Population** | **Domain** | **Recurrent service component** | **Documents endorsing, n/N (%)** | **Strong/mandatory documents** | **Moderate/recommended documents** | **Weak/optional documents** | **Endorsed but not graded documents** |
| --- | --- | --- | --- | --- | --- | --- | --- |
| **FEP** | Service configuration and organization | Specialized outpatient EIP teams | 20/22 (90.9) | MHS Catalunya; SIGN; NICE CG 155; RANZCP; Orygen; CPA FEP; RCPsych; APA; VA-DoD | ISS-SNLG; NICE CG 178 |  | NICE QS 80; HSE; IEPA; IRIS; APP; MMHPI; NCCMH-NICE; MH Ontario; EASA |
| **FEP** | Service configuration and organization | Timely and equitable access to care | 15/22 (68.2) | NICE CG 155; RANZCP; VA-DoD | ISS-SNLG; NICE CG 178; RCPsych | MHS Catalunya; Orygen | NICE QS 80; HSE; IEPA; IRIS; MH Ontario; EASA |
| **FEP** | Service configuration and organization | Integration and coordination with child/adolescent, adult, emergency, and inpatient services | 15/22 (68.2) | NICE CG 178; NICE CG 155; VA-DoD | ISS-SNLG; RCPsych; Orygen | SIGN; RANZCP | HSE; IEPA; IRIS; NCCMH-NICE; MH Ontario; EASA |
| **FEP** | Service configuration and organization | Assertive community treatment and/or intensive case management | 16/22 (72.7) | SIGN; RANZCP; APA; VA-DoD | ISS-SNLG; NICE CG 178; RCPsych | MHS Catalunya; Orygen | NICE QS 80; HSE; IEPA; IRIS; APP; NCCMH-NICE; MH Ontario |
| **FEP** | Service configuration and organization | Inpatient care when clinically indicated | 9/22 (40.9) | NICE CG 178; NICE CG 155 | RCPsych | MHS Catalunya; Orygen | HSE; CAMH; MMHPI; VA-DoD |
| **FEP** | Service configuration and organization | Youth-friendly, non-stigmatizing settings | 14/22 (63.6) | NICE CG 155; CPA FEP | ISS-SNLG; NICE CG 178; RCPsych | MHS Catalunya; RANZCP; Orygen | HSE; IEPA; IRIS; NCCMH-NICE; MH Ontario; EASA |
| **FEP** | Service configuration and organization | Structured policies for transition from youth to adult services | 9/22 (40.9) | NICE CG 155 | RCPsych | RANZCP | HSE; IEPA; IRIS; NCCMH-NICE; MH Ontario; EASA |
| **FEP** | Assessment | Comprehensive multidisciplinary assessment at service entry | 19/22 (86.4) | MHS Catalunya; SIGN; NICE CG 178; NICE CG 155; RANZCP; CPA FEP; RCPsych; APA | ISS-SNLG | Orygen | NICE QS 80; HSE; IEPA; IRIS; APP; MMHPI; NCCMH-NICE; MH Ontario; EASA |
| **FEP** | Assessment | Routine assessment of comorbid psychiatric and substance-use disorders | 15/22 (68.2) | NICE CG 178; NICE CG 155; APA | RANZCP; RCPsych; VA-DoD | SIGN; Orygen | HSE; IEPA; IRIS; NCCMH-NICE; MH Ontario; EASA |
| **FEP** | Assessment | Standardized assessment of functioning and disability | 15/22 (68.2) | NICE CG 155; RCPsych; APA | NICE CG 178; CPA FEP; VA-DoD | RANZCP; Orygen | HSE; IEPA; IRIS; NCCMH-NICE; MH Ontario; EASA |
| **FEP** | Assessment | Validated diagnostic or symptom-rating instruments for diagnosis and monitoring | 16/22 (72.7) | NICE CG 178; NICE CG 155; RCPsych; VA-DoD | MHS Catalunya; SIGN; APA | RANZCP; Orygen | IEPA; HSE; IRIS; NICE QS 80; MH Ontario; EASA; MMHPI |
| **FEP** | Assessment | Regular structured review of physical health parameters | 9/22 (40.9) | NICE CG 155; APA | NICE CG 178 | MHS Catalunya; Orygen | CAMH; HSE; NCCMH-NICE; MH Ontario |
| **FEP** | Treatment | Initiation of antipsychotic medication at low dose with gradual titration | 15/22 (68.2) | MHS Catalunya; NICE CG 178; NICE CG 155; RANZCP; RCPsych; VA-DoD |  | SIGN; Orygen | HSE; IEPA; IRIS; NICE QS 80; APP; NCCMH-NICE; MH Ontario |
| **FEP** | Treatment | Systematic physical health and metabolic monitoring | 21/22 (95.5) | ISS-SNLG; MHS Catalunya; SIGN; NICE CG 178; NICE CG 155; RANZCP; RCPsych; APA |  | Orygen; VA-DoD | NICE QS 80; CAMH; HSE; IEPA; IRIS; APP; MMHPI; NCCMH-NICE; MH Ontario; EASA; ANEP |
| **FEP** | Treatment | Timely consideration of clozapine in treatment-resistant cases | 14/22 (63.6) | ISS-SNLG; MHS Catalunya; NICE CG 178; NICE CG 155; RANZCP; RCPsych; APA; VA-DoD | SIGN |  | HSE; IEPA; IRIS; APP; MMHPI |
| **FEP** | Treatment | CBT-informed psychological interventions for psychosis | 20/22 (90.9) | MHS Catalunya; SIGN; NICE CG 178; NICE CG 155; RANZCP; Orygen; CPA FEP; RCPsych; APA; VA-DoD | ISS-SNLG |  | NICE QS 80; HSE; IEPA; IRIS; APP; MMHPI; NCCMH-NICE; MH Ontario; EASA |
| **FEP** | Treatment | Structured family interventions | 16/22 (72.7) | NICE CG 178; NICE CG 155; RANZCP; RCPsych | CPA FEP; APA | Orygen | MHS Catalunya; SIGN; HSE; IEPA; IRIS; VA-DoD; NICE QS 80; APP; MMHPI |
| **FEP** | Treatment | Psychoeducation for service users and families | 22/22 (100.0) | MHS Catalunya; SIGN; NICE CG 155; RANZCP; Orygen; CPA FEP; APA; VA-DoD; RCPsych | NICE CG 178 | ISS-SNLG | NICE QS 80; ANEP; CAMH; HSE; IEPA; IRIS; APP; MMHPI; NCCMH-NICE; MH Ontario; EASA |
| **FEP** | Treatment | Supported employment and education | 18/22 (81.8) | SIGN; NICE CG 178; NICE CG 155; RANZCP; CPA FEP; RCPsych; APA | MHS Catalunya | Orygen; VA-DoD | HSE; IEPA; IRIS; APP; NCCMH-NICE; MH Ontario; EASA; NICE QS 80 |
| **FEP** | Treatment | Cognitive remediation for persistent cognitive deficits | 9/22 (40.9) | MHS Catalunya; RANZCP | SIGN; CPA FEP; APA | Orygen; VA-DoD | APP; MMHPI |

*This table presents the complete document-level matrix for recurrent FEP service components, showing which included guidance documents endorsed each component and how each endorsement was classified within the harmonized recommendation-strength framework (strong/mandatory, moderate/recommended, weak/optional, or endorsed but not graded). It provides the document-level basis underlying the aggregate synthesis reported in Table 3.*
