# Supplementary material for: Clinical practice guidelines and quality standards for early intervention in psychosis: an AGREE II appraisal and systematic review of service components
Source: Front Psychiatry. 2026 Jun 3;17:1831668. doi: 10.3389/fpsyt.2026.1831668 (PMC13272451; doi:10.3389/fpsyt.2026.1831668)
Supplement: Supplementary file 6 [file Table6.docx]

**Supplementary Table S5. Complete search strategies for each database and grey literature source.**

| **Source** | **Source type** | **Search string / browsing approach** | **Date** | **Records retrieved** |
| --- | --- | --- | --- | --- |
| MEDLINE via PubMed | Database | ("psychosis"[MeSH Terms] OR "psychotic disorders"[MeSH Terms] OR "first episode psychosis"[tiab] OR "first-episode psychosis"[tiab] OR "early psychosis"[tiab] OR "ultra-high risk"[tiab] OR "clinical high risk"[tiab] OR "CHR-P"[tiab] OR "at-risk mental state"[tiab] OR "attenuated psychosis syndrome"[tiab] OR "prodromal psychosis"[tiab]) AND ("practice guideline"[Publication Type] OR "guidelines as topic"[MeSH Terms] OR "clinical practice guideline*"[tiab] OR "quality standard*"[tiab] OR "recommendation*"[tiab] OR "consensus statement*"[tiab]) AND ("early intervention"[tiab] OR "youth mental health"[tiab] OR "young adult*"[tiab] OR "adolescent*"[MeSH Terms] OR "service delivery"[tiab] OR "specialized team*"[tiab] OR "assertive community"[tiab]) | March 2025 | 2,645 |
| Cochrane Library (CENTRAL) | Database | ("psychosis" OR "psychotic disorder*" OR "first episode psychosis" OR "first-episode psychosis" OR "early psychosis" OR "ultra-high risk" OR "clinical high risk" OR "CHR-P" OR "at-risk mental state" OR "attenuated psychosis syndrome" OR "prodromal psychosis") AND ("practice guideline*" OR "clinical practice guideline*" OR "quality standard*" OR "recommendation*" OR "consensus statement*") AND ("early intervention" OR "youth mental health" OR "young adult*" OR "service delivery" OR "specialized team*" OR "assertive community") | March 2025 | — |
| Web of Science (Core Collection) | Database | TS=("psychosis" OR "psychotic disorder*" OR "first episode psychosis" OR "first-episode psychosis" OR "early psychosis" OR "ultra-high risk" OR "clinical high risk" OR "CHR-P" OR "at-risk mental state" OR "attenuated psychosis syndrome" OR "prodromal psychosis") AND TS=("practice guideline*" OR "clinical practice guideline*" OR "quality standard*" OR "recommendation*" OR "consensus statement*") AND TS=("early intervention" OR "youth mental health" OR "young adult*" OR "service delivery" OR "specialized team*" OR "assertive community") | March 2025 | — |
| NICE Evidence Search (evidence.nhs.uk) | Grey literature | Search terms: early intervention psychosis; clinical high risk psychosis; first episode psychosis guidelines. Manual browse of mental health section. | March 2025 | — |
| Guidelines International Network (g-i-n.net) | Grey literature | Manual browse: mental health / psychosis category. | March 2025 | — |
| TRIP Medical Database (tripdatabase.com) | Grey literature | Search terms: early intervention psychosis guidelines; CHR-P guidelines. | March 2025 | — |
| Scottish Intercollegiate Guidelines Network (sign.ac.uk) | Grey literature | Manual browse: mental health and behavioural conditions. | March 2025 | — |
| RANZCP (ranzcp.org) | Grey literature | Manual browse: clinical guidelines. | March 2025 | — |
| Orygen (orygen.org.au) | Grey literature | Manual browse: resources and guidelines. | March 2025 | — |
| International Early Psychosis Association (iepa.org.au) | Grey literature | Manual browse: resources. | March 2025 | — |
| Early Psychosis Intervention Ontario (epion.ca) | Grey literature | Manual browse. | March 2025 | — |
| NHMRC Australia (nhmrc.gov.au) | Grey literature | Manual browse: mental health guidelines. | March 2025 | — |
| Citation chasing | Supplementary | Forward and backward citation searching of all included documents and key systematic reviews in the field. | March 2025 | — |

*Total records identified: 2,778, including 2,645 from MEDLINE (via PubMed) and 133 from non-MEDLINE sources combined. Non-MEDLINE sources included CENTRAL, Web of Science, targeted grey-literature searches, and citation chasing. Source-specific retrieval counts for non-MEDLINE sources were not consistently retained prior to deduplication and are therefore not reported separately. No date filter was applied at the database level beyond the 2005 lower limit; language eligibility was applied at the screening stage. “—” indicates sources for which retrieval counts were not reported separately.*
