# Supplementary material for: Clinical practice guidelines and quality standards for early intervention in psychosis: an AGREE II appraisal and systematic review of service components
Source: Front Psychiatry. 2026 Jun 3;17:1831668. doi: 10.3389/fpsyt.2026.1831668 (PMC13272451; doi:10.3389/fpsyt.2026.1831668)
Supplement: Supplementary file 7 [file Table7.docx]

**Supplementary Table S6. Full-text reports retrieved for eligibility assessment and subsequently excluded, with primary reason for exclusion (PRISMA 2020 item 16b).**

| **Author(s) / Organization** | **Title / Document** | **Year** | **Primary reason for exclusion** |
| --- | --- | --- | --- |
| World Health Organization | mhGAP Intervention Guide for Mental, Neurological and Substance Use Disorders in Non-Specialized Health Settings – Version 2.0 | 2016 | A – general mental health guide without a dedicated CHR-P or FEP care pathway; does not constitute an EIP-specific CPG or QS |
| National Institute for Health and Care Excellence | Psychosis with coexisting substance misuse: assessment and management in adults and young people (CG120) | 2011 | A – focused on dual diagnosis management; no EIP service model or CHR-P pathway |
| American Academy of Child and Adolescent Psychiatry | Practice parameter for the assessment and treatment of children and adolescents with schizophrenia | 2013 | A – paediatric schizophrenia management guideline; no EIP service model or CHR-P pathway |
| National Institute for Health and Care Excellence | Psychosis and schizophrenia in adults: treatment and management – full technical guideline underpinning CG178 | 2014 | A – full technical report underlying NICE CG178 (ref 21); the summary CPG was included; the technical document was not separately coded as an additional included document |
| Galletly C, Castle D, Dark F, et al. (RANZCP) | Royal Australian and New Zealand College of Psychiatrists clinical practice guidelines for the management of schizophrenia and related disorders – 2005 edition | 2005 | B – superseded by the 2016 RANZCP update (ref 26) retained in the review |
| Canadian Psychiatric Association | Clinical practice guidelines for the treatment of schizophrenia | 2005 | B – superseded by the 2017 CPA guidelines (refs 37, 47) retained in the review |
| Orygen Youth Health | Clinical practice guidelines for early psychosis – 1st edition | 2005 | B – superseded by the 2010 2nd edition (ref 54) retained in the review |
| IRIS Initiative Ltd | IRIS guidelines for early intervention in psychosis | 2000 | B – superseded by the 2012 IRIS update (ref 46) retained in the review; also predates the 2005 inclusion threshold |
| NHS England / National Collaborating Centre for Mental Health | A practical guide to implementing the early intervention in psychosis access and waiting time standard | 2016 | B – superseded by the 2023 NCCMH–NICE update (ref 51) retained in the review |
| Addington J, et al. / Canadian Psychiatric Association | Canadian guidelines for the pharmacological treatment of schizophrenia spectrum disorders in children and youth – pre-2017 version | 2009 | B – superseded by the 2017 CPA update (ref 22) retained in the review |
| **[Aggregate] Multiple authors / organizations** | **462 additional full-text reports excluded for reason A: did not constitute a clinical practice guideline or quality standard relevant to early intervention in psychosis per the prespecified eligibility criteria (e.g., treatment guidelines for established schizophrenia without an EIP focus, systematic reviews, editorials, service descriptions, conference abstracts, locally adapted guidance without formal endorsement). Individual citations available from the corresponding author on request.** | **Various** | **A – did not constitute an EIP-relevant CPG or QS (n = 462)** |
| **[Aggregate] Multiple authors / organizations** | **84 additional full-text reports excluded for reason B: documents superseded by updated versions retained in the review, or limited to a single comorbidity or care setting without a broader EIP care pathway. Individual citations available from the corresponding author on request.** | **Various** | **B – superseded or partial scope (n = 84)** |

*Total full-text reports assessed for eligibility: 582. Total excluded: 556. Included: 26. Exclusion reason A = did not constitute a clinical practice guideline or quality standard relevant to early intervention in psychosis per the prespecified inclusion criteria. Exclusion reason B = superseded by a more recent version from the same issuing body retained in the review, or limited to a single comorbidity or setting without broader EIP care pathway recommendations. Where a report met more than one criterion, the primary reason is recorded.*
